# Brain-wide mapping reveals temporal and sexually dimorphic opioid actions

**DOI:** 10.1038/s42003-026-09730-8

**Published:** 2026-02-20

**Authors:** Iaroslavna Vasylieva, Reese Smith, Eshan Aravind, Lora L. Pless, Kelin He, Tianhan Ling, Jenesis Kozel, Stephanie Puig, Katarzyna M. Kedziora, Jessica J. Scarlett, Paul N. Joseph, Matthew D. Lycas, Benjamin R. Williams, Mackenzie C. Gamble, Ulrik Gether, Ryan W. Logan, Zachary Freyberg, Alan M. Watson

**Affiliations:** 1 Center for Biologic Imaging, University of Pittsburgh, Pittsburgh, PA USA; 2 Department of Cell Biology, University of Pittsburgh, Pittsburgh, PA USA; 3 Department of Psychiatry, University of Pittsburgh, Pittsburgh, PA USA; 4 Department of Medicine, Division of Infectious Diseases, University of Pittsburgh, Pittsburgh, PA USA; 5 Department of Psychiatry and Behavioral Sciences, University of Massachusetts Chan Medical School, Worcester, MA USA; 6 Department of Neurobiology, University of Massachusetts Chan Medical School, Worcester, MA USA; 7 Department of Neuroscience, University of Copenhagen, Copenhagen, Denmark; 8 Molecular and Translational Medicine, Department of Medicine, Boston University School of Medicine, Boston, MA USA; 9 Department of Pharmacology, Biochemistry & Biophysics, Boston University School of Medicine, Boston, MA USA

**Keywords:** Computational neuroscience, Experimental models of disease, Computational neuroscience, Fluorescence imaging

## Abstract

The field of neuroscience has been transformed by recent advances in spatial mapping of neuronal activity across whole cleared brains. Rapid adoption of these techniques requires computational workflows that can facilitate experiments comparing multiple conditions across large cohorts of individuals. We therefore developed a scalable approach for anatomical mapping of c-Fos positive cells in whole brain and applied it to map the response to the prototypic opioid, morphine. The analysis revealed distinct patterns of morphine-induced regional brain activation across both time and sex. These results support the multi-wave model of opioid-induced brain activation. Male mice displayed higher c-Fos expression than females in several key brain regions including nucleus accumbens, central amygdalar nucleus, ventral pallidum, prelimbic area, anterior cingulate area, and olfactory tubercle. Overall, this workflow can be applied to not only examine spatiotemporal actions of drugs of abuse on neuronal activity across the brain, but also mapping neuronal activity more generally.

## Abstract (web summary)

This study introduces a semi-automated, scalable whole-brain mapping workflow that enables unbiased comparisons across cohorts, revealing widespread temporally and sexually distinct regional activation patterns after acute morphine exposure in mice.

## Introduction

Opioids are some of the most widely used and abused substances today, playing a central role in the treatment of pain. However, these drugs can also induce tolerance and dependence, significantly contributing to their high potential for abuse^1^. As a result, the prevalence of opioid abuse has skyrocketed both in the United States and worldwide^2–4^. Current pharmacological treatments for opioid use disorder (OUD) are effective in mitigating cravings, although 90% of patients relapse within several months of treatment cessation^5^. Thus, to identify novel treatments for OUD and interventions capable of slowing the opioid epidemic, we must elucidate the neurobiology of opioid addiction and its relationships to factors that contribute to craving and relapse vulnerability.

An initial and acute exposure to opioids can lead to rapid adaptive changes in the brain that reflect the early mechanisms of opioid dependence and the risk of subsequent addiction. Indeed, treating humans or rodents with opioid antagonists can elicit signs of withdrawal^6^. Changes in neuronal activity across various populations of neurons occur following acute administration of opioids, likely underlying the adaptive changes that contribute to tolerance and withdrawal^7–10^. Revealing *where* these changes in activity occur, and their relationship to drug-related behaviors, is critical for understanding the fundamental actions of drugs of abuse, including opioids, and for providing potential neurobiological targets for therapeutic intervention. Consequently, the neuroscience field has long grappled with how to effectively investigate activity across multiple scales within the same brain, from the single cell to ensembles to entire brain regions, and *how* drugs modulate these changes in activity.

The biological importance of neuronal heterogeneity in addiction and the need for scalability have been widely acknowledged. However, most current approaches are still limited by their ability to resolve distinct changes in activity in response to drugs of abuse at the single-cell level within larger populations throughout the whole brain. For example, immunolabeling of neuronal activity markers across various brain regions has been valuable in revealing new neurobiological substrates of complex drug-related behaviors^7,9,11,12^. Yet, because of constraints of throughput and resolution, earlier work typically focused on investigating discrete regions in areas previously implicated in addiction. As a result, these prior limitations created a bottleneck for the development of a more integrated understanding of the biological underpinnings of addiction at a whole-brain level. The ability to widely survey the whole brain at sufficient resolution allows us to move beyond single brain regions. Rather, we can begin examining opioid actions across multiple regions—a key next step in the generation of a holistic, more integrated understanding of drug actions.

Whole-brain mapping of neuronal activation in cleared tissue is becoming state-of-the-art in rodent preclinical studies^13–15^. Immediate early genes (IEG) such as *c-Fos* allow for the rapid detection of brain neuronal activity in response to a variety of stimuli. *c-Fos* is widely used in studies of social behaviors^16^, effects of stress^17^, drug treatment^18^, and addiction^19^. As the use cases for mapping neuronal activity grow, scientists will need to contrast multiple experimental conditions with large numbers of individuals to reach statistically relevant sample sizes. Although existing workflows for whole-brain *c-Fos* mapping have progressively become more accurate and broadly applicable^13,20^, these methods are generally not designed to scale across dozens of brains and finish within time frames amenable to the iterative nature of hypothesis-driven research. For example, studies of opioid addiction require large numbers of brains to address variables including time, treatment, and sex, in addition to compensating for the high variability inherent among animals.

To address the above challenges, we have developed a workflow that offers the capability to readily move across scales throughout whole intact brains in three dimensions (3D). Rather than being limited to a single brain region or smaller subsets of neurons, newer whole-brain imaging and analysis methods provide the opportunity to probe drug-induced effects in an unbiased manner. Here, we used tissue clearing^21^ and high-speed ribbon scanning confocal microscopy^22^ (RSCM) to collect high-resolution imagery of whole brains. RSCM allows mounting of multiple brains at once and automatic acquisition of the images without manual intervention. Confocal microscopy is also more tolerant to tissue clearing imperfections than light-sheet approaches that require very good clearing^23,24^, making this method compatible with more clearing methods. We applied a novel high-performance computing workflow that detects and maps cells within the brain. The development of this integrated workflow allowed us to digitally reconstruct entire adult mouse brains at sub-cellular resolution and therefore to comprehensively map activated neurons across the entire brain in dozens of animals. Using this approach to data analysis, we can tackle experimental questions like time-, sex-, and brain region-dependent differences in opioid actions.

Opioid response has been shown to be sexually dimorphic in analgesia and addiction^25,26^. Temporally, opioids cause two waves of IEG expression: an early wave (~30 m post injection) that mainly activates basal ganglia, and a broader late wave (~6 h post injection) that activates the cortex^27^. While the structures activated shortly after injection are well characterized, 1h and 4h time points are relatively understudied. By 4h post-drug exposure, injection-related stress returns to baseline^28^, and analgesic effects dissipate, providing an opportunity to more clearly examine plasticity-related changes that emerge during the late wave. Sex differences have similarly been understudied at the 1 h and 4 h time points, with prior work mainly focused on long-term effects of drug exposure. Altogether, this creates a strong rationale to examine opioid actions across the whole brain in male and female mice at 1h and 4h time points.

In the present study, we utilized the developed workflow to comprehensively characterize the global anatomical expression patterns of *c-Fos* in response to the administration of the prototypical opioid, morphine. Our data demonstrated broad patterns of increased activity within the brains of morphine-exposed wild-type mice. The patterns of morphine-induced activity demonstrated time- and sex-specific differences in neuronal activity in male and female animals. In summary, our imaging and analysis approaches open new avenues for answering long-standing questions in opioid actions, including resolving cellular heterogeneity within larger populations and mapping their changes in response to drug exposure.

## Results

### Development of a whole-brain imaging and analysis workflow

**Fig. 1:**
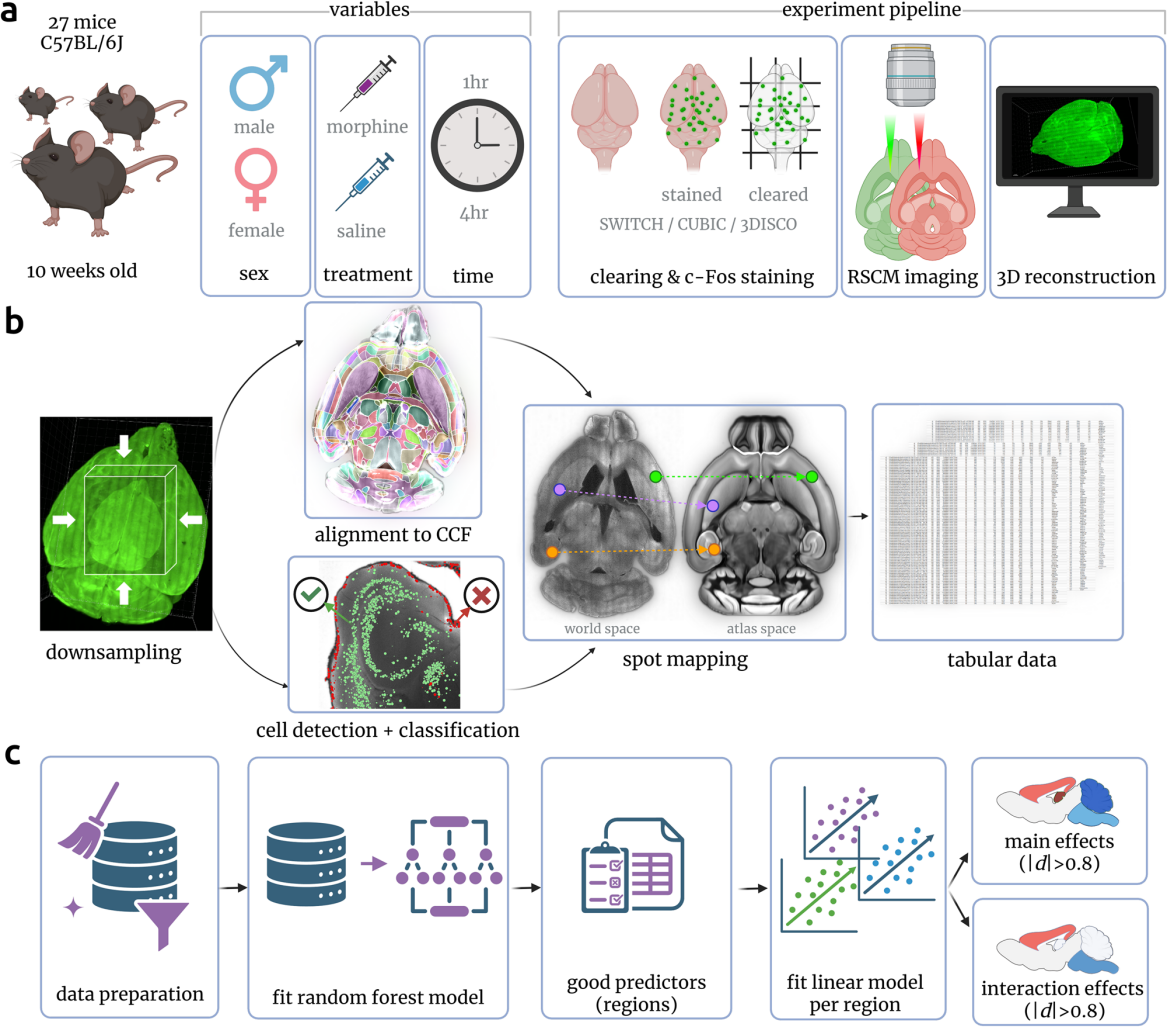
Experimental design. **a** 10-week-old male and female mouse were injected with morphine or saline. 1h or 4h post injection, their brains were harvested, stained for c-Fos, and rendered optically transparent. Cleared brains were imaged using RSCM and then reconstructed in 3D. The 3D images were run through the quantitative data analysis pipeline (**b**). The 3D brain reconstructions were first downsampled to ~10 μm resolution. Images were subsequently registered to the CCFv3. In parallel, c-Fos-positive cells were detected, and each location was recorded in the 3D dataset (world space). Each spot was then classified as cell/non-cell using a neural network. Coordinates for each cell were transformed to the CCFv3 space using the deformation field obtained from the image registration procedure and associated with a CCFv3 brain region (spot mapping). The resulting tables of spot centroids and corresponding brain regions were aggregated by brain region and across treatment groups for statistical analysis. **c** Data was organized into the wide format to fit the random forest model, which identified brain regions contributing to morphine response (good predictors). Then, a linear model with treatment, time, and sex as factors was fit to the data from regions pre-selected by the random forest model. Created in BioRender. Vasylieva, I. (2026) https://BioRender.com/elabc2b, https://BioRender.com/f50zcz1.

**Fig. 2:**
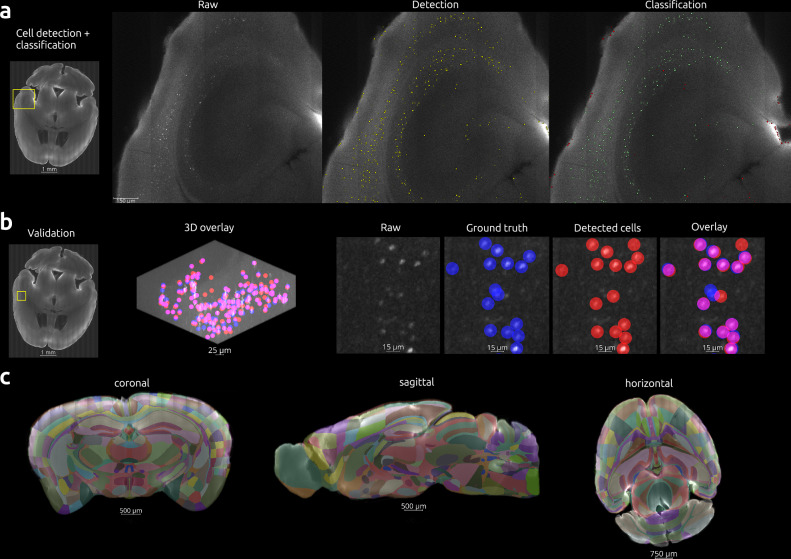
Example of whole brain analysis including cell detection, classification, and atlas alignment. An example brain from the Morphine/Female/4h group is depicted. **a** Raw imaging data were used for automated detection of cells (yellow spots) as described in the methods. Neural network classification determined whether each spot was a cell (green) or non-cell (red). All spots determined to be cells were used for downstream analysis. An ROI **b** of size 250 × 250 × 25 voxels was manually annotated for ground truth (blue) by an expert annotator and used to validate the automated cell counting and classification approach (red). F-1 score for this region was 93.5%. Intersecting spots (purple) were considered to represent the same cell. The 3D projection displays all cells within the ROI (left). Since the location of each spot was determined by a centroid indicating the center of the detected cell, minor variations between ground truth and automated detection are seen as lateral offsets. A 5-micron radius from the centroid was used to determine whether spots intersected. The 2D data depicted are ~20 μm maximum intensity projections of a zoomed-in region within the larger 3D projection and display spots where the centroids were located within the dimensions of the projection. Each centroid was located within the CCFv3 following alignment as depicted in **c** and as described in the methods.

To investigate the impact of different durations of opioid exposure on the activities of cell subpopulations throughout the whole adult mouse brain, we administered morphine to male and female wild-type mice versus a vehicle control (Fig. 1a). 1 h or 4 h following exposure, brains were collected and fluorescently stained for c-Fos, followed by optical clearing and imaging via RSCM. After imaging, data from each brain were processed through a novel analysis pipeline (Fig. 1b) consisting of parallel steps for down-scaling the data for alignment to the Allen Mouse Brain Common Coordinate Framework version 3 (CCFv3^29^) and detection of c-Fos-positive cells throughout the brain (Fig. 2a, c). We used a combination of conventional and deep-learning spot detection approaches, followed by clustering duplicate detections and neural network-based discrimination of true cells and artifacts.

All aspects of the computational workflow were completed semi-autonomously on local high-performance computing resources managed by the Simple Linux Utility for Resource Management (SLURM^30^). Following RSCM, data were automatically queued for stitching and assembly into 3D datasets. After manual verification of each 3D dataset, spot detection, alignment, and classification were queued as a single job, outputting intermediate processing products such as the alignment deformation field and finally the spot tables which included CCFv3-mapped structures. Wherever possible, we reused already available open-source toolkits, developing novel means to knit the software together to produce an automated workflow requiring minimal intervention. Following an initial pass, the results of each alignment and cell classification were manually reviewed and, when appropriate, reprocessed with modified parameters. Although we used SLURM to manage this process, the workflow can be run on a single mid- to high-end workstation with a NVIDIA GPU, making it accessible to most research laboratories.

Performance of automated spot detection and classification was measured against a ground truth data sample collected for each brain (Figs. 2b and S3). The acceptance criterion was an f1-score of >80%. For the brain having a lower f1-score, the base classification model was fine-tuned to reach the 80% threshold. For each brain, the coordinates of c-Fos-positive cells were transformed into CCFv3 space and exported as CSV files that would be the basis for all subsequent data mining. During analysis, we employed an unbiased approach by inspecting morphine’s effects on every brain structure within the gray matter, at every depth level in the CCFv3 structure tree. This included calculating the total numbers and densities of c-Fos-positive cells for each respective brain structure. We fitted a random forest machine learning model to identify which brain regions were good predictors of morphine treatment. We then used the selected regions to fit a linear mixed model and identify morphine actions as well as time and sex differences in them (Fig. 1c).

### Morphine induces broad increases in c-Fos activity across the whole brain

**Fig. 3:**
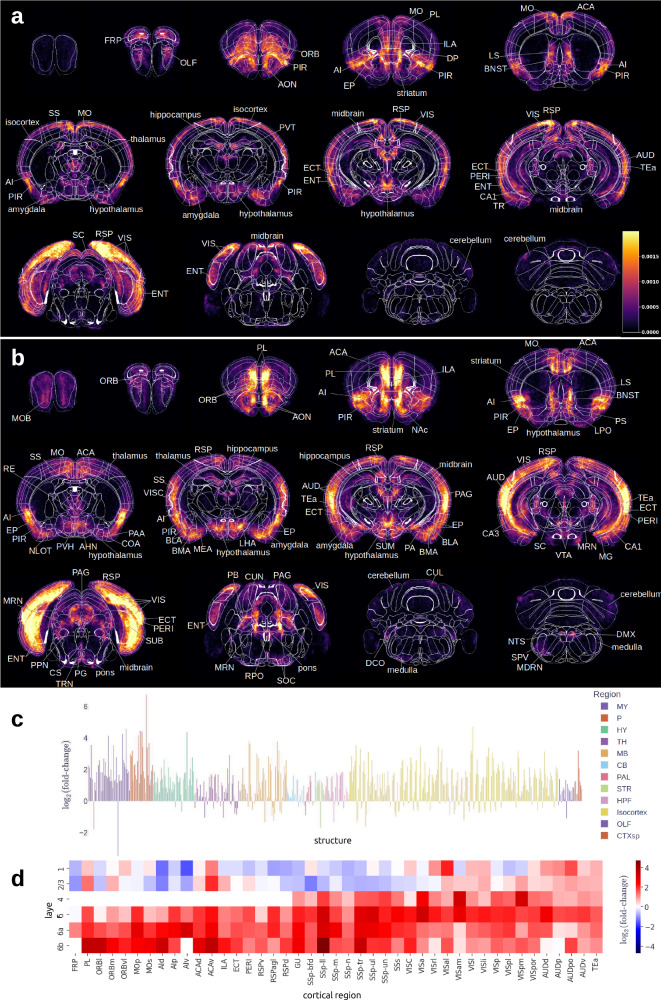
Global c-Fos expression in mice exposed to morphine. 1 mm thick coronal heatmaps* of c-Fos positive neurons for all **a** saline and **b** morphine treated animals. Areas containing the brightest signal are labeled according to the CCFv3 structure. Morphine/saline c-Fos density fold-change is displayed for all grey matter brain structures **c** within the CCFv3 and grouped by larger brain region as indicated by color. Cortical areas **d** are grouped by layer with color representing log_2_(fold-change). Blue indicates depressed activity in a region due to morphine exposure, and red indicates enhanced activity. *Heatmaps in panels a and b were calculated by aggregating all c-FOS positive neurons, dividing by the total number of animals, and then smoothing with a Gaussian kernel. All images are displayed on the same scale (CCFv3). Abbreviations used can be found at atlas.brain-map.org.

**Fig. 4:**
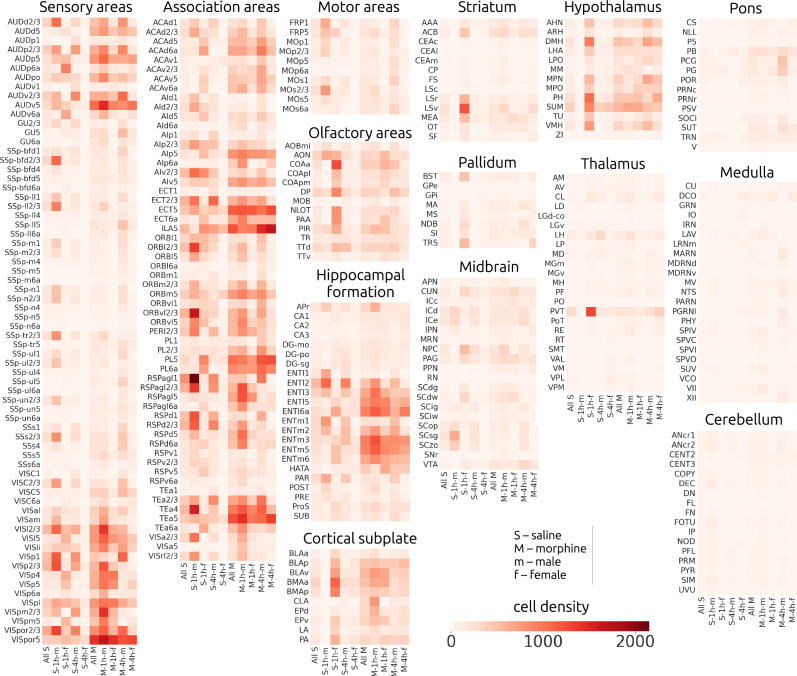
c-Fos-positive cell densities. A heat map displaying the density of c-Fos expressing cells (cells/mm^3^) organized according to experimental groups and arranged according to prominent brain structures. Structures were selected based on their depth in the CCFv3 structure tree (leaves, deepest structures) and volume > 0.25 mm^3^. Experimental groups were the smallest sets of animals that differed by all 3 variables (treatment, time-point, sex). There were 8 experimental groups: morphine-1h-male (M-1h-m, *N* = 5), morphine-1h-female (M-1h-f, *N* = 3), morphine-4h-male (M-4h-m, *N* = 4), morphine-4h-female (M-4h-f, *N* = 3), saline-1h-male (S-1h-m, *N* = 3), saline-1h-female (S-1h-f, *N* = 2), saline-4h-male (S-4h-m, *N* = 3), saline-4h-female (S-4h-f, *N* = 4). Abbreviations used can be found at atlas.brain-map.org.

To determine whether morphine exposure induced measurable changes in c-Fos expression across the mouse brain, we plotted coronal heatmaps of the average cellular density of c-Fos-positive cells from saline- (Fig. 3a) and morphine-exposed (Fig. 3b) animals at 1mm projections. There were observable elevations in c-Fos-positive cell density across the brains of morphine-exposed animals with qualitatively evident increases in the cortical layers. Quantitative analysis confirmed that more c-Fos-positive cells were present in brains of morphine-exposed mice compared to brains of saline-treated controls (average densities 234 ± 36 cells/mm^3^ and 140 ± 43 cells/mm^3^, respectively; *p* = 0.1). This increase in morphine-induced cell activity was observed across most brain regions (Fig. 3c) and remained consistent overall when examining individual experimental groups (Fig. 4).

The Allen Mouse Brain Atlas is organized as a hierarchical ontology, where broad anatomical divisions are recursively subdivided into finer regions, nuclei and layers. This tree-like structure provides parent-child relationships used for consistent multiscale annotation across experiments. At a coarser scale, we examined large brain divisions that collectively constitute 90% of the brain volume. Our data showed that each of these structures, on average, had more c-Fos-positive cells in brains of morphine-exposed animals compared to saline controls (Table 1 and Fig. S1). Morphine-exposed brains demonstrated the greatest c-Fos+ density fold-change in pons and medulla, and the lowest fold-change in the thalamus and cerebellum. Statistically significant increases at this depth of the anatomical hierarchy were only observed in the cortical subplate and hypothalamus. Overall, the highest density of morphine-activated c-Fos puncta was observed in the cortical subplate and isocortex. In many cases, large anatomical brain structures contained subregions that exhibited opposing changes in c-Fos signal. For example, the Isocortex showed mostly increased signal in layers 4-6, and decreased signal in cortical layers 1-3 (Fig. 3d). However, layers 1-3 displayed increased c-Fos signal in regions associated with auditory and visual processing.

**Table 1:** Morphine effect on major brain structures: Cortical subplate (CTXsp), Striatum (STR), Thalamus (TH), Hypothalamus (HY), Pallidum (PAL), Hippocampal formation (HPF), Pons (P), Medulla (MY), Olfactory areas (OLF), Midbrain (MB), Isocortex and Cerebellum (CB)

Structure	Density *M*	Density *S*	*d*	*d*_*lower*_	*d*_*upper*_	*p*	*q* (FDR)	significant
CTXsp	463.94	212.38	2.53	1.29	3.76	0.0001	0.0008	***
STR	119.69	71.30	1.59	0.20	2.99	0.0249	0.0808	
TH	63.82	52.47	1.31	0.15	2.47	0.0269	0.0808	
HY	331.58	163.64	1.15	0.40	1.90	0.0028	0.0167	*
PAL	121.84	74.60	1.04	−0.07	2.14	0.0652	0.1565	
HPF	393.18	215.53	1.03	−0.39	2.45	0.1548	0.2954	
P	121.47	32.28	0.84	−0.38	2.06	0.1772	0.2954	
MY	48.93	17.46	0.79	−0.41	1.99	0.1969	0.2954	
OLF	329.66	222.46	0.77	−0.47	2.02	0.2238	0.2983	
MB	180.87	119.48	0.72	−0.66	2.09	0.3078	0.3693	
Isocortex	417.89	256.76	0.10	−0.88	1.07	0.8464	0.8464	
CB	49.68	39.40	−0.22	−1.58	1.15	0.7534	0.8219	

Columns: CCFv3 structure, average c-Fos+ density across morphine brains, average c-Fos+ density across saline brains, Cohen’s *d*, lower limit of *d* confidence interval, upper limit of confidence interval, uncorrected *p*-value, FDR corrected *q*-value, significance marked using standardized star notation (*q < 0.05, ***q < 0.001). Sorted by *d*.

Using the random forest model’s feature importance, we identified at least 359 of 722 gray matter structures that contributed to the overall brain response to morphine exposure. Notably, among structures previously shown to respond to morphine, all had nonzero importance and ranked high in the feature importance table (e.g., periaqueductal grey (PAG) ranked #28 of 722 structures, dorsal Raphe nucleus (DR) ranked #16, nucleus accumbens (ACB) ranked #138, ventral tegmental area (VTA) ranked #177, etc.). Contributing structures contained both parent and child regions, which would likely have a correlated c-Fos expression and introduced multicollinearity into the model. We therefore excluded parent structures if their children were on this list; the remaining 276 child structures were further analyzed statistically.

**Fig. 5:**
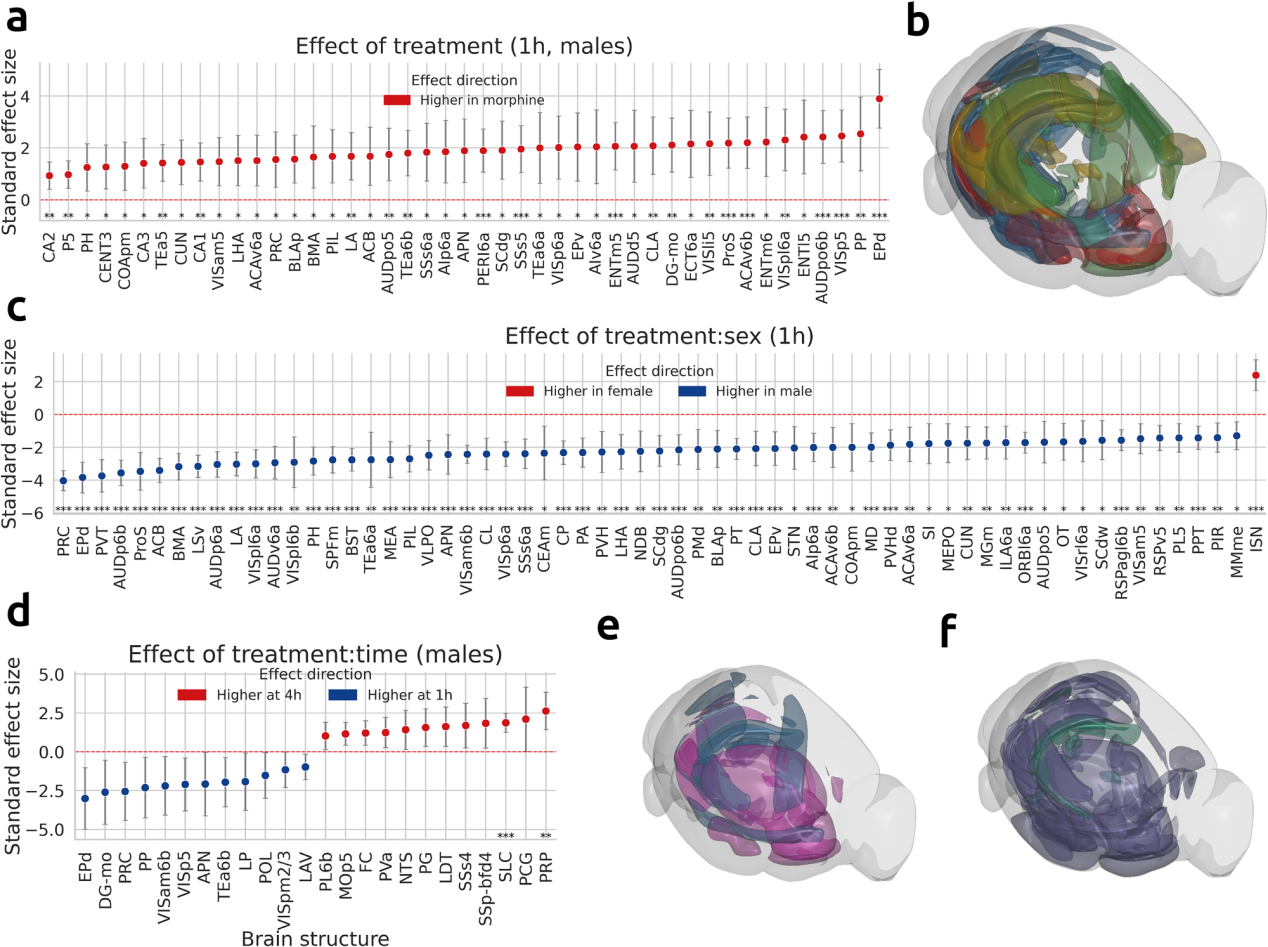
Time- and sex-dependent effects of morphine on region-specific brain activity. **a** Structures that have large morphine effect (Cohen’s ∣*d*∣  > 0.8) at baseline (1h, males). Only structures with *q* < 0.05 are shown. **c** Structures that have sex differences (large interaction effect of treatment:sex) at baseline (1h). Only structures with *q* < 0.05 are shown. **d** Structures that have time differences (large interaction effect of treatment:time) at baseline (males). All structures with ∣*d*∣ > 0.8 are shown. Structures with *q* < 0.05 are marked with standardized star notation (*q < 0.05, **q < 0.01, ***q < 0.001). **b** Structures that show large morphine effect in males at 1h (blue), in males at 4h (green), in females at 1h (red), in females at 4h (yellow) are shown in a 3D brain rendering. **e** Structures that exhibit time differences in males (blue) and females (pink). **f** Structures that exhibit sex differences at 1h (purple) and at 4h (green). Effect sizes for regions having large effects not at baseline are plotted in Fig. S4. Abbreviations used can be found at atlas.brain-map.org.

We next conducted statistical analyses using linear models with treatment, time, and sex as factors. Our results revealed 132 structures that responded to morphine (Table 2 and Figs. 5a, and S4a–c). We considered a structure responding to morphine if it had a large main effect of treatment (Cohen’s ∣*d*∣ > 0.8) in at least one of the morphine groups (Male-1h, Male-4h, Female-1h, or Female-4h), and the confidence interval of this effect did not contain 0. Most of the structures that responded to morphine (n=58) were located in isocortex, and included subregions of anterior cingulate area (ACA), prelimbic area (PL), temporal association area (TEa), visceral area (VISC), ectorhinal area (ECT), retrosplenial area (RSP), infralimbic area (ILA), and agranular insular area (AI). In addition to cortical regions, morphine-responsive structures included subregions of every large brain counterpart from Table 1. The majority of these structures (*n* = 82) were contributed by Male-1h group (Table S1). The Female-1h group contributed 25 structures partially overlapping with the Male-1h group (Table S3); Male-4h and Female-4h contributed 37 and 17 structures, respectively (Table S2, S4). Corrections for multiple comparisons involving hundreds of brain regions may be too stringent on subtle effects; therefore, we report the effect sizes and confidence intervals for all structures showing large effects, whereas statistically significant structures are marked using standard star notation in Figs. 5 and S4.

**Table 2:** Brain regions responding to morphine

Major Structure	Count	Morphine responsive subregions
Isocortex	58	ACAd6a, ACAv2/3, ACAv5, ACAv6a, ACAv6b, AIp6a, AIv6a, AUDd5, AUDp6a, AUDp6b, AUDpo5, AUDpo6b, AUDv5, AUDv6a, ECT6a, ILA6a, MOp5, MOs6a, ORBm6a, PERI5, PERI6a, PL5, PL6a, PL6b, RSPagl1, RSPagl6a, RSPv6a, SSp-bfd5, SSp-bfd6b, SSp-n1, SSp-ul4, SSp-ul5, SSp-un4, SSp-un5, SSs4, SSs5, SSs6a, TEa5, TEa6a, TEa6b, VISC5, VISa5, VISam5, VISam6b, VISl6b, VISli5, VISli6a, VISp5, VISp6a, VISp6b, VISpl5, VISpl6a, VISpl6b, VISpm5, VISpor5, VISpor6a, VISpor6b, VISrl5
CTXsp	7	PA, BLAp, LA, CLA, EPd, BMA, EPv
HPF	12	CA1, FC, ProS, CA3, ENTl6a, ENTm5, CA2, DG-mo, HATA, ENTm6, ENTl5, ENTl3
OLF	1	COApm
HY	9	MMme, MPN, PH, VLPO, PVH, LHA, PVHd, PMd, PVa
TH	10	PIL, PP, MGm, VPMpc, PT, PoT, SPFm, PVT, CL, POL
MB	14	IPI, PRC, DR, CUN, IPRL, LT, SCdg, MT, IF, VTA, RR, SNr, PPN, APN
P	8	LDT, DTN, PG, PCG, SLC, P5, SUT, SLD
MY	4	PRP, NTS, ISN, DMX
STR	4	CEAm, MEA, LSv, ACB
PAL	3	BST, SI, NDB
CB	2	CENT2, CENT3

Organized by major brain structures belonging to: Isocortex, Cortical subplate (CTXsp), Striatum (STR), Thalamus (TH), Hypothalamus (HY), Pallidum (PAL), Hippocampal formation (HPF), Pons (P), Medulla (MY), Olfactory areas (OLF), Midbrain (MB), and Cerebellum (CB). Abbreviations used can be found at atlas.brain-map.org.

### Acute exposure to morphine induces rapid changes in c-Fos activity in regions implicated in addiction

Among morphine-responding regions, we found classical structures implicated in analgesia and different stages of opioid addiction cycle (not only the acute “binge" stage) (Table 2). Our method detected VTA—a midbrain region enriched in dopaminergic neurons; nucleus accumbens (ACB), a key striatal region to which midbrain neurons project and which is implicated in drug reward; substantia innominata (SI, ventral pallidum), which is downstream of the ACB; and dorsal raphe nucleus (DR)—a serotonergic region. We also identified precommissural nucleus (PRC), a subregion of PAG, which is responsible for opioid-induced analgesia, and relaying nuclei of thalamus (paraventricular, PVT, posterior intralaminar, PIL, ventral posteromedial, VPMpc) and medulla (nucleus of solitary tract, NTS). Moreover, we identified structures implicated in drug withdrawal including the central nucleus of the amygdala (CEA), bed nucleus of the stria terminalis (BST)^31–33^, along with other regions in the extended amygdala (lateral, LA, posterior, PA, and basomedial, BMA amygdalar nuclei), hypothalamic regions (paraventricular, PVH, and lateral, LHA hypothalamus) and insula (AI). Finally, we identified structures crucial in later drug preoccupation/anticipation stages, including basolateral maygdala (BLA), hippocampus (CA1, CA2, CA3), and subregions of the prefrontal cortex (prelimbic, PL, anterior cingulate, ACA, and infralimbic, ILA areas), also exhibited large effects.

### Different sets of brain regions respond to morphine at 1h and 4h

We determined the impacts of different durations of morphine exposure on brain region activation. Fewer brain regions responded to morphine at 4h than at 1h: 53 versus 95 structures, respectively, with 16 structures shared between the time points. Moreover, there were slightly fewer c-Fos puncta at 4h overall (density 254 ± 62 at 1h vs 212 ± 37 at 4h). All large effects that we observed at 4h were positive (activating). We found a two-wave pattern of c-Fos activation similar to other studies^27^. In our case, morphine-responsive regions in the 1h group included structures implicated in mediating analgesia and reward, while the activated structures within the 4h group extended more broadly to cortical regions, spanning subregions of the ACA, PL, ILA, somatosensory (SS), somatomotor (MO), and orbital (ORB) areas. Interestingly, the 4h group also showed activation in the cortical regions albeit in subregions that differed from those activated at 1h post-morphine exposure including subregions of auditory (AUD), visual (VIS), agranular insular (AI), retrosplenial (RSP), entorhinal (ENTl), and temporal association (TEa) areas. Our analysis likely reflects the transition from subcortical opioid-output circuits at 1h to cortical-hippocampal plasticity circuits at 4h.

To assess how time modulated the brain response to morphine statistically, we examined treatment:time interactions (Figs. 5d, e and S4e). The treatment:time interaction had a large negative effect (decrease from 1h to 4h) in 12 structures at baseline (males, Table S5). This included several subcortical regions important for analgesia and locomotion, and corresponded to the early wave of opioid-induced activation, caused by immediate functional effects of opioids. For example, the dorsal endopiriform nucleus (EPd), a deep-layer piriform/claustral interface that receives dense cortical and limbic inputs and is a component of the early motivational/salience circuitry, showed rapid c-Fos induction after acute morphine exposure. We similarly identified activation in the anterior pretectal nucleus (APN), a midbrain motor and antinociceptive relay responsive to opioids and pain modulation early after exposure. Finally, we observed activation of the lateral posterior thalamic nucleus (LP) and posterior limiting nucleus of the thalamus (POL), areas of early thalamocortical sensory relay response.

We also found 12 structures with large positive treatment:time interaction effect, where treatment with morphine demonstrated an increase in c-Fos expression from 1h to 4h in males (baseline) and 23 structures in females (Fig. S4e and Table S6). The PL, MO, and SS cortical layers show delayed c-Fos induction consistent with late cortical plasticity. Prosubiculum (ProS) and Fasciola cinerea (FC) are hippocampal regions responsible for plasticity crucial for contextual memory of drug exposure. Periventricular hypothalamic (PVa) and thalamic (PVT) nuclei are morphine-sensitive and control endocrine and arousal regulation. PRC, subregion of PAG, and pontine central gray (PCG) are parts of the brainstem-(PAG)-pontine continuum, which is a classic site of opioid analgesia. All structures that exhibited time differences are summarized in Table 3.

**Table 3:** Time and sex differences in response to morphine

Major structure	Time differences	Sex differences
Isocortex	AUDp6a, AUDp6b, AUDv6a, ILA6a, MOp5, PL6b, SSp-bfd4, SSs4, TEa6b, VISam6b, VISl6b, VISp5, VISp6b, VISpl6b, VISpm2/3	ACAv6a, ACAv6b, AIp6a, AUDd5, AUDp6a, AUDp6b, AUDpo5, AUDpo6b, AUDv4, AUDv6a, ILA1, ILA6a, ORBl6a, ORBm6a, PL5, RSPagl6a, RSPagl6b, RSPd6a, RSPv5, SSs6a, SSs6b, TEa6a, VISam5, VISam6b, VISl6b, VISp6a, VISp6b, VISpl6a, VISpl6b, VISrl5, VISrl6a
CTXsp	BMA, EPd	BLAp, BMA, CLA, EPd, EPv, LA, PA
HPF	DG-mo, FC, ProS	CA1, DG-mo, ENTm5, ProS
OLF	-	COApl, COApm, PIR
HY	PH, PVa, VLPO	AVP, LHA, MEPO, MMme, MPN, PH, PMd, PVH, PVHd, STN, SUM, VLPO
TH	CL, LP, POL, PP, PVT, SPFm, VPMpc	CL, IMD, MD, MGm, PIL, PT, PVT, SPFm, VPMpc
MB	APN, IF, PRC	APN, CUN, IPI, PPT, PRC, SCdg, SCdw
P	LDT, PCG, PG, SLC	DTN, LDT, PCG, SLD
MY	LAV, NTS, PRP	ISN, LRNm, PRP
STR	ACB, CP, LSv	ACB, CEAm, CP, LSv, MEA, OT
PAL	BAC, BST, SI	BST, NDB, SI
CB	-	-

Organized by major brain structure: Isocortex, Cortical subplate (CTXsp), Striatum (STR), Thalamus (TH), Hypothalamus (HY), Pallidum (PAL), Hippocampal formation (HPF), Pons (P), Medulla (MY), Olfactory areas (OLF), Midbrain (MB) and Cerebellum (CB). Abbreviations used can be found at atlas.brain-map.org.

### Male and female mice display distinct patterns of c-Fos expression in response to morphine

We examined whether the response to morphine differed based on sex. Male mice showed more c-fos expression after morphine treatment, both at 1h and 4h following exposure (Fig. S2). For male mice, the effect of morphine was mostly activating both at 1h and 4h. In contrast, in female mice, morphine’s effects on c-Fos expression were inhibitory at 1h and activating at 4h. We posit this may be due to the high values in the corresponding saline controls caused by females’ stronger reactions to stress shortly after injection, consistent with the literature^34^. The positive treatment:time interaction effects in females confirmed that c-Fos expression increased by 4h (Fig. S4e).

Our analysis revealed 80 structures displaying large treatment:sex interactions (sex differences, Fig. 5c, f and Table S7) at baseline (1h). Most of them showed negative effect, i.e., males showed a larger morphine-evoked change in c-Fos expression compared to females. More neuronal activation in males than females fits a general picture of stronger *μ*-opioid receptor (MOR) coupling in males^26,35^. The structures with sex differences included regions classically implicated in addiction and analgesia: nucleus accumbens, where there is evidence of sex differences in dopamine release following opioids in models of opioid self-administration^36^, and the lateral septum (LS), where MOR binding is reported to differ by sex^37^; PRC, a part of the PAG. We also detected sex differences in structures that are implicated in withdrawal/negative affect stage, including most amygdalar regions and BST, which has rich *κ*- and *μ*- opioid receptor (KOR, MOR) signaling and is implicated in stress-addiction interactions with emerging sex-specific findings^38^. Paraventricular thalamus (PVT), a region connected to stress and reward circuits, has evidence of sex-specific adaptations after opioid abstinence in rats^39^. Ventrolateral preoptic nucleus (VLPO) controls sleep, and sex effects on opioid modulation of sleep are increasingly noted^40,41^. Claustrum (CLA) was recently reported to be implicated in opioid relapse^42^ and fentanyl intake control^43^. Olfactory tubercle (OT) is a reward hub with opioid-reward links, but there’s little information on sex differences. Finally, among the structures normally associated with the preoccupation/anticipation stage, we detected sex differences in hippocampal dentate gyrus (DG) and cornu ammonis (CA), where opioids alter plasticity and receptor distribution in a sex-specific manner^44^. There were fewer sex differences detected at 4h; only 10 structures (Table S8) exhibited large tratment:time effects, including substructures within the pons and medulla (Fig. S4d). All brain regions showing sex differences are summarized in Table 3.

## Discussion

In our study, we present a fast, high-throughput approach for mapping drug-evoked neuronal activity in the brains of mice acutely exposed to morphine. Our approach combined high-speed microscopic imaging techniques, high-performance computing workflows, and validated open-source software tools to identify and map cells within the whole mouse brain. We collected high-resolution whole-brain data across sexes and times after morphine exposure. Our workflow was designed around integrating validated open-source software tools into a novel pipeline that were able to be run on a high-end workstation, but could be deployed on a high-performance computing infrastructure for scalability. Existing whole brain mapping workflows like ClearMap^13^ and BrainGlobe^45^ are themselves not explicitly designed as integrated distributed workflows. However, components of these ecosystems could be adapted for use in our pipeline. Indeed, our pipeline used BrainGlobe’s BrainMapper for cell detection and Brainreg for atlas alignment. For cell detection, we used two independent detection approaches (BrainMappter and deepblink), and their results were consolidated using DBSCAN for 3D clustering. Cell classification is build around the BrainMapper approach, but we re-implemented it to improve the speed of training and inference. There are other promising methods that could replace steps in this workflow, such as cellpose^46^ and recent spotiflow^47^. However, cellpose is primarily used for high-magnification 2D cell images. Both of these methods would need to be retrained for use with our data. We expect that future improvements to the workflow may include integration of other excellent open-source tools for brain alignment^48–50^, cell detection^20,47^, and classification^51^ as needed.

Although our pipeline was designed for minimal user interaction, approximately 30% (8/27) of our samples required manual interventions. Several samples had lighting artifacts or a strong striped pattern; therefore, pre-processing was needed for them to achieve satisfactory alignment. Due to poor contrast, for some samples, we needed to fine-tune the classification neural network. These issues might be avoided by using a different imaging modality like light-sheet. However, our choice of RSCM enabled multiple samples to be mounted and queued for imaging simultaneously, and we expect that imaging systems that can only mount single samples would impact throughput. In the future, appropriate AI image restoration methods and more robust alignment tools may reduce the need for these manual interventions.

Our workflow revealed time-, region-, and sex-specific differences in c-Fos activity throughout discrete regions of the whole brain. This includes changes in areas of the brain associated with the reward circuitry^32^ and more broadly, the cortex-basal ganglia loop involved in reinforcement learning and choice of behavioral programs^52^. Moreover, male and female animals displayed differences in morphine responses, with males displaying increased activity overall compared to females. This included higher activation in brain regions that belong to the mesolimbic dopamine system^53^, broader reward circuits^32^, and the extended amygdala^54^ implicated in addiction, including nucleus accumbens, ventral pallidum, central amygdala, basolateral amygdala, olfactory tubercle, prelimbic area, and cingulate cortex. Collectively, our work opens the door to a robust, unbiased approach to identify novel drug-induced temporal and spatial changes in whole-brain activity at cellular resolution.

We found that data from many brain regions is required to reliably predict the effects of morphine vs saline treatment across the whole brain. A random forest model trained on all 722 gray matter regions achieved higher accuracy (81%) compared to a model trained on a subset of structures that included only leaf-level regions from the Allen Atlas tree (74%). The random forest model allowed us to get a broad picture of regions responding to morphine prior to fitting a linear model on relevant regions. Such a strategy enabled us to delineate the influence of time and sex. Statistical analysis revealed region-specific neuronal activation in response to morphine treatment across 132 brain structures. Although our data confirmed activity in previously reported brain regions^55^, we also found unexpected morphine effects in regions not traditionally studied in the context of opioid actions. We can divide all regions we found in 3 tiers, from the ones that are classically studied in the context of opioid addiction, to the ones not mentioned in the literature. The first tier included ACB, VTA, PRC/PAG, central amygdalar nucleus (CEAm), other amygdalar regions, BST, PVT, PVH, LHA, and NTS. The second tier is a group of regions that are also associated with opioid actions via receptors, peptides, or functional studies: cortico-limbic, decision-making and emotion control regions (PL, ACA, ILA, ORB, AI^56,57^), hippocampal regions associated with memory and learning (CA1-3, DG, hippocampo-amygdalar transition area (HATA), ProS,^58^), extended limbic system (ventral pallidum, lateral septum, diagonal band nucleus (NDB)), raphe nuclei (dorsal (DR), interfascicular^59^), thalamic (medial geniculate complex (MGm), ventral posteromedial, posterior triangular, subparafascicular (SPFm), posterior intralaminar (PIL), peripeduncular, parataenial (PT)^60,61^), hypothalamic (medial mammillary (MMme), medial preoptic (MPN), ventrolateral preoptic, periventricular^62–64^) and brainstem (laterodorsal tegmental, pedunculopontine, subceruleus (SLC), peritrigeminal, dorsal motor vagus nerve^65,66^) nuclei. Finally, the third tier is the regions mentioned in the opioid literature only indirectly (e.g., in receptor atlases^67^) or not mentioned at all. These include ectorhinal, perirhinal, temporal, and entorhinal association cortical regions, parts of RSP, SS, MO, VIS and AUD cortices, multiple subcortical regions (POL, APN, cuneiform nucleus (CUN), superior colliculus (SCdg), posterior hypothalamic nucleus (PH)), brainstem regions (pontine gray, pontine central gray, sublaterodorsal nucleus, supratrigeminal nucleus, dorsal tegmental nucleus), and central cerebellar lobules (CENT2, CENT3)^68–71^.

Notably, our data are consistent with previous work showing that many of the morphine-responsive regions also express MORs according to a brain atlas of MOR expression^67^. While MORs are G_*αi*/*o*_ G protein-coupled receptors (GPCRs) that typically inhibit neuronal activity via hyperpolarization and suppression of neurotransmitter release, morphine-induced c-Fos activation in these regions may reflect indirect or circuit-level effects rather than direct MOR-mediated stimulation. Consistent with this, opioids can modulate the pain circuitry both directly and indirectly, such as through actions on serotonergic neurons that project from the PAG through the rostral ventromedial medulla en route to the spinal cord^72^. These potential MOR-serotonin links are also evident in the prominent morphine-induced activation of the DR and related raphe nuclei, structures classically associated with the serotonin system, which is in line with previous research^72–76^.

We identified distinct sex differences in the response to morphine in male versus female mice, consistent with growing evidence of sex differences associated with opioid actions both clinically and in preclinical models^26,77^. In preclinical models, females demonstrate greater opioid self-administration compared to males^78–81^. Likewise, clinically, women are 4-fold more likely to inject heroin compared to men^25,82^. Interestingly, we found that males exhibited more extensive morphine-induced cell activation in the striatum, pallidum, amygdala, and hippocampus. Regions like PAG, ACB, hippocampus, amygdala, and PVT are known to have sex differences in response to opioids. Sex differences in PAG, where males have higher activation than females, are consistent with prior work due to lower MOR density and coupling efficacy in females and therefore lower disinhibition of PAG output neurons^83^. The same is true for the nucleus accumbens (ACB)^84^, where we observed less c-Fos expression in females. Similarly, in amygdala, lateral (LA), basolateral (BLA), central (CEA), medial (MEA), and posterior (PA) nuclei show higher c-Fos upregulation in males, presumably due to males exhibiting higher MOR density, and females having stronger estrogenic modulation that reduces inhibition of GABAergic interneurons^85,86^. Other regions we found to have sex differences include APN, MPN, LHA, PVH, SPFm, interpeduncular nucleus, and posterior pretectal nucleus, which are nodes that participate in opioid modulation of aversion, pain, arousal, and locomotion^87–91^. While these brain regions have been implicated in opioid actions, evidence of potential sex differences is sparse. No prior literature on sex differences in opioids explicitly mentions NDB, PT, PIL, ProS, CUN, MGm, SCdg, SCdw, PH, PT, MMme, subthalamic nucleus, mediodorsal and intermediodorsal nuclei of thalamus, dorsal premammillary nucleus, median and anteroventral preoptic nuclei of hypothalamus, piriform area, endopiriform nuclei (EPd, EPv), caudoputamen, supramammillary nucleus, inferior salivatory nucleus, and cortical amygdalar area (COApm, COApl).

Interestingly, the dorsal endopiriform nucleus (EPd) shows strong activation and a strong sex difference in our data. A study characterizing a KOR knock-in mouse line found relatively high KOR expression in the dorsal endopiriform nucleus^92^, although there are no detailed studies of MOR functional activation nor sex differences in EPd. This raises the possibility that the above sex differences in morphine-induced brain region activation are due to sexually dimorphic patterns of opioid receptor expression. It is also possible that these sex differences are at least partially mediated by estrogen, given estrogen’s emerging role as a modulator of the brain opioid system^25,86^. Future work will be required to elucidate the mechanisms underlying the interactions between opioid actions, sex, and brain regions.

We have also found temporal differences in cell activation at 1h versus 4h time points. At 1h post-exposure, 45 brain regions had significant differences between morphine and saline in males (*q* < 0.05), and 10 regions in females, after correction for multiple comparisons. 37 and 15 more regions (*q* ≥ 0.05), respectively, had a large effect of morphine (∣*d*∣ > 0.8), both positive and negative. Positive effects are likely the downstream result of disinhibition, whereas negative effects could be explained either by inhibition of neurons with postsynaptic opioid receptors by morphine (e.g., PRC and PVT that had a negative effect in females) or a high saline background shortly after injection. At 4h post-exposure, despite the absence of brain region differences that remained statistically significant after correction for multiple comparisons, there were regions showing large positive effects of morphine both in males and females. There were no regions with large negative effects at 4h. Our findings are therefore in agreement with previous studies showing that c-Fos expression peaks in both morphine and saline brains at 30–60 m post-injection, followed by a return to baseline in saline but not morphine conditions^27^.

Only subceruleus (SLC) and prepositus (PRP) brainstem nuclei showed significant positive treatment:time interaction (q<0.05) in males, and PVT - in females. PVT was inhibited at 1h, likely due to both inhibition of neurons with postsynaptic opioid receptors (*μ* and *κ*) and modulation of inhibitory input onto PVT neurons presynaptically^93^, and then had a positive treatment:time interaction effect, i.e., the neuronal firing recovered by 4h. SLC is a continuation of locus ceruleus (LC) in the rodent brainstem, and is part of the same noradrenergic complex, which is strongly modulated by opioid receptors^94^ Therefore, it is likely to be inhibited at 1h, and increase activation toward 4h. PRP is an enkephalinergic input to LC^95^, however, it does not stand out as a site of opioid receptors.

We note that our use of c-Fos labeling to identify morphine-induced neuronal activity may capture the strongest stimuli but still miss other groups of cells with less robust patterns of activation^96^. This can sometimes be exacerbated by the inherent properties of the brain tissue and imperfections in clearing, which lead to light absorption and scattering, ultimately reducing signal-to-noise deep within the tissue. To increase our chances of detecting low signal-to-noise events, our workflow leveraged two methods of cell detection, combining conventional threshold-based detection and neural networks that are threshold independent. While c-Fos detection acknowledges activity-driven expression, this approach does not permit measurement of the magnitude of cell activation. Thus, we cannot use our approach to quantitatively ascertain whether already-activated cells exhibit a change of expression in response to different durations of morphine exposure.

Our data suggest that manipulation of the mice (e.g., injection-related stress) may impact brain region activation independently of the drug treatments. Indeed, prior work showed that stress-induced activation of brain regions resembled patterns we identified in our study^28^. Group housing and returning the mice from 4h-groups to their home cage after 1h are possible factors that could impact c-Fos expression. We accounted for this by manipulating both experimental and control groups identically. Future studies will quantify this stress/manipulation effect using a control group with no manipulation at all.

Very small brain regions require exceedingly accurate alignment. Inaccuracies in the multimodal alignment of large volumetric datasets are to be expected. Modality-specific atlas reference images^97^ exist for other imaging modalities and might improve accuracy, but currently there is no confocal-specific template for the CCFv3. In our experience, several advanced image alignment methods (ANTS^48^, LDDMM^49^, NiftyREG^98^) yielded similar results. Future improvements in multi-modal alignment may improve accuracy. We expect that small inconsistencies in alignments would average out with greater numbers of animals per group. Thus, we expect that future studies will focus on imaging a greater number of animals, which will enable us to obtain both increased accuracy and statistical power, and account for high between-subject variability.

### Conclusions

Recent technological advances in tissue clearing and whole-brain imaging have enabled us to digitally reconstruct entire mouse brains at cellular resolution. By merging high-speed RSCM^22^ and high-performance computing workflows designed to detect and map individual cells to locations within the brain, we can comprehensively map drug-activated neurons across many animals in an unbiased manner at single-cell resolution. The resulting data demonstrate broad patterns of increased activity within the brains of morphine-exposed animals in a sex- and region-specific manner. Importantly, a single experiment allowed us to confirm the role of structures described in a diverse literature focused on specific brain regions and simultaneously identify novel structures that vary with time and sex. Overall, our fast whole-brain imaging combined with massively parallel analysis opens avenues for exploring a large parameter space, including actions of different drugs, different time courses of drug action, as well as sex- and region-specific effects. These studies, therefore, offer a more comprehensive understanding of the actions of opioids and offer targets for the development of novel therapeutic approaches.

## Methods

### Animals

Adult wildtype male and female mice (C57BL/6J, Jackson Laboratory, stock # 000664) were used throughout the experiment (Total *N* = 27; 15 males, 12 females, 8–10-weeks-old). All animals were housed in a 12/12 light cycle (7 a.m. lights on, 7 p.m. lights off). Water and rodent chow were provided ad libitum throughout the experiment. Animals were habituated to the laboratory environment for 1 week prior to experimental testing. We have complied with all relevant ethical regulations for animal use. All procedures received ethical approval and were performed in compliance with the Institutional Animal Care and Use Committee at Boston University and the University of Pittsburgh.

### Drugs

All drugs were obtained from Sigma-Aldrich (St. Louis, MO) unless otherwise noted. Morphine sulfate was dissolved in a solution of filtered (0.22 μM) 0.9% saline.

### Drug treatment and brain collection

Mice were randomly assigned to 1h or 4h treatment groups. Animals were injected i.p. with either morphine (10 mg/kg) or saline (10 mg/kg, w/v). Morphine solution or the saline vehicle solution was injected intraperitoneally (i.p.) in awake animals that were lightly restrained. Mice were immediately placed in a novel environment consisting of a plexiglass chamber with animal bedding. Animals were allowed to explore this environment for 1h before undergoing brain collection (1h group) or returned to their home cage for 3h before brain collection at the 4h time point (4h group). All mice were group housed before and after the 1h time point. Animals underwent transcardiac perfusion with ice-cold PBS until blood had been cleared from the mouse. This was followed by 4% paraformaldehyde (PFA) perfusion for 10 m. Fixed brains were dissected and then transferred to 4% PFA at 4 ^∘^C for 24h before transferring to ice-cold PBS.

### Tissue clearing and staining

Brains were prepared using a mixture of CUBIC^99^, 3DISCO^100^, and SWITCH^101^ clearing and staining protocols. All steps took place at room temperature. The samples were pre-cleared using 50% CUBIC R1 for 24h then 100% CUBIC R1 for 1–2weeks, changing the solution every 24-48h. After pre-clearing, CUBIC R1 was rinsed out, and samples were stained with a rabbit monoclonal anti-phospho-c-Fos primary antibody (catalog# 8677S, Cell Signaling Technology, Danvers, MA) according to the SWITCH protocol. Samples were equilibrated in SWITCH-Off solution (5 mM SDS + 0.04% sodium azide) for 24h, followed by the addition of the anti-c-Fos primary antibody (Rb) to the samples (1:250 in SWITCH-Off solution). Samples were incubated in primary antibody for 1 week, then washed for 24–48h in SWITCH-On solution (1X PBS + 0.04% sodium azide + Triton-X 100). This process was repeated with Cy5-conjugated goat and donkey anti-rabbit secondary antibodies (1:2000, catalog numbers 111-605-003 and 711-175-152, Jackson Immunoresearch, West Grove, PA) in SWITCH-Off. Samples were post-fixed in 4% paraformaldehyde for 1h. After staining, samples were dehydrated in 2-h cycles of increasing concentrations of tetrahydrofuran (%THF: 30, 50, 70, 90 (x2), 100 (x2)). Following dehydration, samples were cleared and mounted in dibenzyl ether prior to imaging. Brains with substantial tissue damage or insufficient staining penetration were discarded.

### Imaging

Brains were imaged via ribbon scanning confocal microscopy (RSCM, CALIBER I.D. RS-G4, Andover, MA, USA) as described previously^22^. Briefly, up to 8 brains were immersed in dibenzyl ether and arrayed in a single imaging chamber. The chamber was sealed with a glass coverslip and then filled with glycerol. We used a Nikon 20x, 1.0NA, 8.2 mm WD, glycerol immersion (CF190) objective specifically designed for cleared tissue imaging. Data were acquired using a 488 (autofluorescence filter Semrock FF01-520/44) and 647 (Cy5 c-Fos signal filter Semrock FF01-670/30) filter with full sequencing enabled. Four samples were imaged at 0.337μm × 0.337 μm × 4.57 μm (X,Y,Z) voxel resolution, and twenty-three samples were imaged with 0.361 μm × 0.361 μm × 5.33 μm (X,Y,Z) voxel resolution. The data were stitched and assembled into 2D images, each representing a single channel and z-plane. Shot noise was then removed from the images using a convolutional neural network based on noise-2-noise^102^ and trained on RSCM data. Image planes were then assembled into a 3D volume using the freely available Imaris File Converter (Bitplane), resulting in a single Imaris file composed of a multiscale chunked data structure that represented the entire whole brain dataset. All subsequent downstream analysis was completed from these files.

### Analysis environment

Image processing was performed using the University of Pittsburgh Center for Biologic Imaging’s high performance computing environment. The environment is schedulable via SLURM and consists of an 18-node compute partition with 24-cores and 96GB RAM, a 5-node GPU partition, each with 72 cores, 1.5TB RAM, and 8x NVIDIA P40, and an AI node with 224-cores, 4TB RAM, and 8x NVIDIA H200. All nodes mount two BeeGFS high-performance file systems consisting of 7PB of HDD and 160TB of SSD. RSCM data was acquired directly as unstitched ribbons to the BeeGFS SSD storage system prior to being stitched and assembled on the compute nodes as 2D images. Data were then copied to the HDD storage system, where they were queued for denoising on the GPU nodes. Following denoising, all images were assembled into Imaris (.ims) files, which were the basis for all remaining image analysis. The final size of each full brain dataset was, on average ~1.5 terabytes, comprising 40TB across 27 brains. During image generation and processing, these data were transformed 3 times transiently, generating approximately 200TB. The final denoised .ims files were retained and are publicly available at the Brain Image Library as discussed below. Data mining and analysis were developed and tested on custom-built workstations consisting of 32-core, 64-thread AMD Ryzen Threadripper PRO, 256GB RAM, and NVIDIA A6000 GPU.

### c-Fos puncta detection

We observed fluorescent c-Fos-positive puncta of different sizes and intensities. We found that the larger and brighter spots were well-detected with the BrainMapper (cellfinder^45^) python tool, whereas small and dim spots were detectable with the deepblink particle model^103^ based on UNet architecture^104^. Both algorithms were applied to each imaged brain. Together, these algorithms were purposely biased towards over-detection to ensure that all cells were identified. Thus, to avoid duplicate detections, the resulting spots were run through DBSCAN clustering algorithm^105^, which clustered the duplicated detections together and replaced these clusters with a single point. In addition, detected points contained artifacts such as edges, noise peaks, and autofluorescent vasculature. To eliminate these artifacts, each detected spot was fed to a deep learning binary classifier based on ResNet-50^106^. The model was trained on >40,000 cell and non-cell examples across multiple brains from the present study. To confirm the precision, recall, and f1^107^ score of the detection and classification process, ground truth data were annotated manually in a small patch of size 25 × 250 × 250 pixels from each brain in a region having a clear c-Fos signal. For brains where the f1 score was <80%, additional improvement of classification was needed. The model was subsequently fine-tuned on a relatively small number of manually annotated data from each of these brains (e.g., 50–200 examples of cells and non-cells), using fastai^108^. Finally, the data was inspected visually, and for a few brains where classification still did not reach a f1 score of ≥80%, minor manual removal of residual artifacts was done. Optional removal of background detections could reduce the time spent on clustering duplicated detections and cell/artifact classification. We created background-foreground masks for each brain using the accelerated pixel and object classification plugin for napari^109^ that employs a random forest machine learning algorithm^110^.

### Alignment to the CCFv3

Each brain was aligned to the 10 μm CCFv3 following downsampling of the full resolution to 10 μm isotropic resolution. Brainreg^111^, a fully automated 3D registration Python package, was used for all alignments with the default parameters. The alignment results were inspected visually. If the alignment quality was not satisfactory, the raw data were pre-processed by filtering in the frequency domain, contrast stretching, and background subtraction until a visually good alignment was obtained. The resulting deformation field was used to transform each of the c-Fos points into CCFv3 space. Every c-Fos-positive cell was associated with an Allen Mouse Brain Atlas parcellation where it was located.

Critically, tissue clearing results in some physical deformation of the mouse brain. Although relatively rare, the stresses placed on delicate brain tissue can sometimes cause tearing or result in tissue loss. Though nonlinear alignment algorithms are used in brainreg as an attempt to correct for changes in tissue shape, they are never entirely accurate. The alignment quality metrics for each brain are given in Table S9. We used the same alignment initialization parameters for each brain.

### Statistics and reproducibility

We obtained the locations of c-Fos-positive cells that mapped to the Allen Mouse Brain CCFv3 and were labeled with the associated parcellation. We collected data that corresponded to the coordinates in the raw brain image (world space), the coordinates for the CCFv3, and the corresponding CCFv3 parcellation (i.e., brain structure). For each brain specimen, we quantified c-Fos-positive cell density per brain structure by dividing the cell count per structure by the volume of that structure. For each brain, the points were aggregated by brain structure, and the density of c-Fos-positive cells for each structure was calculated as the number of spots in the structure over the volume of the structure. We note that the Allen Mouse Brain atlas is left-right symmetric, and the structure labels did not differ between hemispheres; therefore, left and right counterparts of the same structure were combined.

Densities were the main metric used to make comparisons of structures across brains from different animals and different experimental conditions. A random forest model^110^ was first used to identify brain regions that best discriminated between morphine- and saline-treated mice (scikit-learn^112^ implementation). Data was organized in a wide format for analysis. We performed 10-fold cross-validation with 300 estimators (trees) followed by fitting a final model to the full dataset. The random forest model achieved a cross-validated performance of 81% (F1-score). Feature importances were extracted for all regions, and those with a nonzero importance were selected for further analyses.

After selecting relevant brain regions using the random forest model, we fit a linear mixed model (LMM) using statsmodels^113^. The LMM included treatment, time, sex, and their interactions as fixed effects. A random intercept was incorporated per mouse to account for non-independence of repeated measures (brain regions). We extracted fixed effects (mean differences, *β*) from the model, and the standardized effect size (Cohen’s *d*,^114^) with corresponding confidence intervals was calculated using the residual standard deviation and random intercept variance from the model. We report regions with a large effect size, defined with ∣*d*∣ > 0.8, for both main effects and two-way interaction effects. The p-values were corrected for multiple comparisons using Benjamini-Hochberg False Discovery Rate (FDR^115^). Regions that had an FDR *q* < 0.05 were considered significant. Our secondary data tables have been made public alongside the raw data as previously mentioned. Unless otherwise indicated, data are represented as mean ± standard error of the mean. Analyses were conducted using the Python programming language within an interactive Jupyter notebook^116^, unless otherwise specified.

### Visualizations

The raw imaging data were stored in the Imaris file format, and the Imaris viewer (Oxford Instruments, UK) was used for initial visual inspection. Volumetric brain cartoons were made using brainrender^117^. Other charts were done using Plotly^118^ or Seaborn^119^. Heatmaps in the CCFv3 were made in napari^120^.

## Supplementary information

Supplementary Information

Reporting-Summary

## Data Availability

The data described herein have been deposited in the Brain Image Library (BIL^121^) and are available along with the derived analysis data under 10.35077/g.1192.

## Code availability

All code used to generate the results described in this manuscript is available at github.com/CBI-PITT/cfos_morphine_pipeline. Jupyter notebooks are provided, which reproduce the figures and tables.
