# Supplementary material for: Brain-wide mapping reveals temporal and sexually dimorphic opioid actions
Source: Commun Biol. 2026 Feb 20;9:466. doi: 10.1038/s42003-026-09730-8 (PMC13036026; doi:10.1038/s42003-026-09730-8)
Supplement: Supplementary file 1 — Supplementary Information [file 42003_2026_9730_MOESM1_ESM.pdf]

Supplemental Materials for
"Brain-wide mapping reveals temporal and sexually dimorphic opioid actions"

Abbreviations

BIL - Brain Image Library
CCFv3 - Allen Brain Atlas Common Coordinate Framework, Version 3
CSV - Character Separated Values
FAIR - Findable, Accessible, Interoperable, Reusable
GPU - Graphical Processing Unit
HDD - Hard Disk Drive
IEG - Immediate Early Gene
MOR - μ-opoid receptor
OUD - Opioid Use Disorder
PB - Petabyte
RAM - Random Access Memory
ROI - Region of Interest
RSCM - Ribbon Scanning Confocal Microscopy
SLURM - Simple Linux Utility for Resource Management
SSD - Solid State Drive
TB - Terabyte
Brain Regions found in the CCFv3
ACA - Anterior Cingulate Area
ACB - Nucleus Accumbens
AI - Agranular Insular Area
APN - Anterior Pretectal Nucleus
AUD - Auditory areas
AVP - Anteroventral preoptic nucleus
BAC - Bed nucleus of the anterior commissure
BLA - Basolateral amygdalar nucleus
BMA - Basomedial amygdalar nucleus
BST, BNST - Bed Nucleus of the Stria Terminalis
CA - Cornu Ammonis
CB - Cerebellum
CEA - Central amygdalar nucleus
CENT - Central lobule
CL - Central lateral nucleus of the thalamus
CLA - Claustrum
COA - Cortical amygdalar area
CP - Caudoputamen
CUN - Cuneiform nucleus
CTXsp - Cortical Subplate
DG-mo - Dentate Gyrus, molecular layer
DMX - Dorsal motor nucleus of the vagus nerve
DR - Dorsal Raphe Nucleus
DTN - Dorsal tegmental nucleus
ECT - Ectorhinal Area
EP - Endopiriform nucleus
ENT - Entorhinal Area
FC - Fasciola cinerea
HPF - Hippocampal Formation
HATA - Hippocampus-Amygdala Transition Area
HY – Hypothalamus
IF - Interfascicular nucleus raphe
ILA - Infralimbic Area

ISN - Inferior salivatory nucleus
IP - Interpeduncular nucleus
LA - Lateral Amygdalar Nucleus
LAV - Lateral Vestibular Nucleus
LDT - Laterodorsal tegmental nucleus
LHA - Lateral Hypothalamic Area
LP - Lateral Posterior Nucleus
LS - Lateral septal nucleus
LT - Lateral terminal nucleus of the accessory optic tract
LRNm - Lateral Reticular Nucleus, medial part
MEA - Medial Amygdalar Nucleus
MEPO - Median preoptic nucleus
MB - Midbrain
MGm - Medial Geniculate Nucleus, magnocellular division
MM - Medial Mammillary Nucleus
MO - Somatomotor areas
MPN - Medial preoptic nucleus
MT - Medial terminal nucleus of the accessory optic tract
MY - Medulla
NAc - Nucleus Accumbens
NDB - Nucleus of the Diagonal Band
NTS - Nucleus of the Solitary Tract
ORB - Orbital area
OT - Olfactory Tubercle
P - Pons
PA - Posterior Amygdalar Nucleus
PAL - Pallidum
PAG - Periaqueductal Gray
P5 - Peritrigeminal zone
PCG - Pontine Central Gray
PERI - Perirhinal area
PG - Pontine Gray
PH - Posterior hypothalamic nucleus
PIL - Posterior intralaminar thalamic nucleus
PIR - Piriform area
PL - Prelimbic area
PMd - Dorsal premammillary nucleus
POL - Posterior limiting nucleus of the thalamus
PoT - Posterior triangular thalamic nucleus
PP - Peripeduncular nucleus
PPT - Posterior pretectal nucleus
PRC - Precommissural nucleus
ProS - Prosubiculum
PRP - Nucleus prepositus
PPN - Pedunculopontine nucleus
PT - Parataenial nucleus
PV - Periventricular hypothalamic nucleus
PVH - Paraventricular hypothalamic nucleus
PVT - Paraventricular nucleus of the thalamus
RR - Midbrain reticular nucleus, retrorubral area
RSP - Retrosplenial area
SC - Superior colliculus
SI - Substantia innominata
SLC - Subceruleus nucleus
SLD - Sublaterodorsal nucleus

SN - Substantia Nigra
SPF - Subparafascicular nucleus
SS - Somatosensory Area
STN - Subthalamic nucleus
STR - Striatum
SUT - Supratrigeminal Nucleus
SUM - Supramammillary Nucleus
TEa - Temporal Association Area
TH - Thalamus
VISC - Visceral Area
VIS - Visual areas
VLPO - Ventrolateral preoptic nucleus
VTA - Ventral Tegmental Area
VPM - Ventral Posteromedial Nucleus

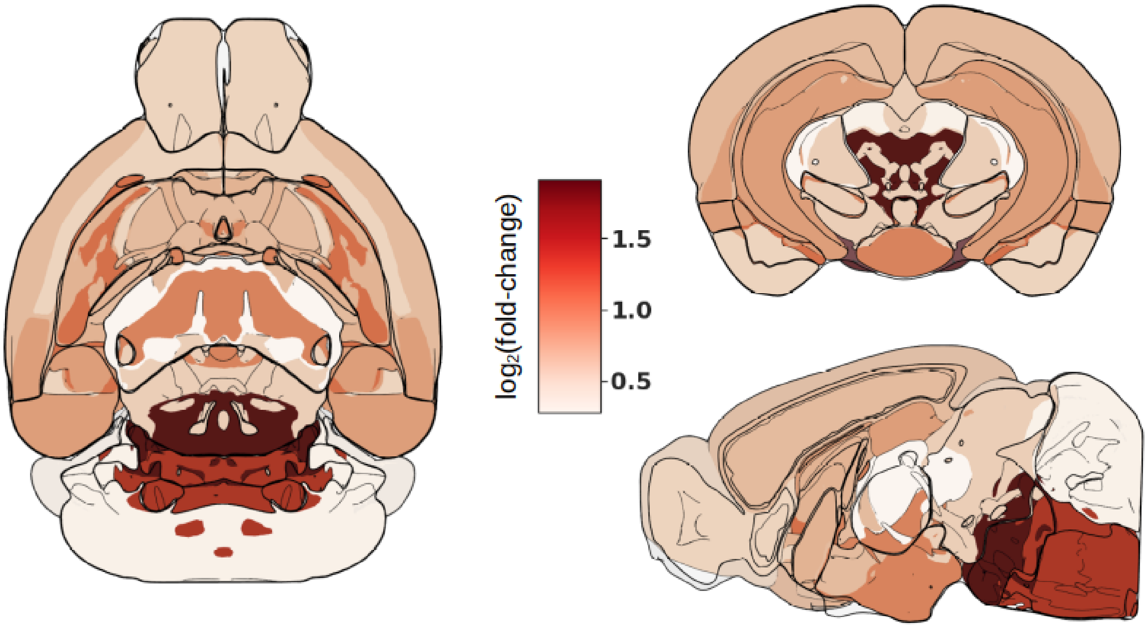

Figure S1. Morphine effect across large brain structures. A cartoon representation of the mouse brain with a heat map which displays the average density fold-change across structures summarized in Table 1. Average morphine cell densities are calculated across all 15 morphine treated brains in the study. Average saline densities are calculated across all 12 saline treated brains.

Table S1. Morphine responsive structures (1h, male, |*d*| > 0.8) Allen Mouse Brain Atlas gray matter structures that exhibited a large morphine effect at baseline (1h, male mice). Sorted by standardized effect size (*d*). Abbreviations used can be found at atlas.brain-map.org.

structure	β	β_{lower}	β_{upper}	d	d_{lower}	d_{upper}	$\lvert d\rvert$	p	q
EPd	750.0	531.9	968.1	3.89	2.76	5.03	3.89	< 0.001	< 0.001

Continued on next page

structure	β	β_{lower}	β_{upper}	d	d_{lower}	d_{upper}	$\lvert d \rvert$	p	q
PP	483.0	212.3	753.7	2.54	1.12	3.96	2.54	0.0005	0.0076
VISp5	1164.0	687.8	1640.1	2.46	1.45	3.46	2.46	< 0.001	0.0002
AUDpo6b	525.4	305.1	745.7	2.42	1.41	3.43	2.42	< 0.001	0.0003
ENTl5	1145.6	473.3	1817.8	2.42	1.00	3.84	2.42	0.0008	0.0105
VISpl6a	877.5	424.9	1330.1	2.30	1.12	3.49	2.30	0.0001	0.0031
ENTm6	1034.2	413.7	1654.6	2.22	0.89	3.56	2.22	0.0011	0.0115
ACAv6b	504.9	277.8	731.9	2.20	1.21	3.19	2.20	< 0.001	0.0005
ProS	350.7	195.1	506.4	2.18	1.21	3.15	2.18	< 0.001	0.0005
VISli5	1352.7	584.6	2120.9	2.16	0.93	3.38	2.16	0.0006	0.0084
ECT6a	1093.0	438.2	1747.7	2.15	0.86	3.44	2.15	0.0011	0.0115
DG-mo	188.4	94.9	281.8	2.11	1.06	3.15	2.11	0.0001	0.0022
CLA	1004.7	468.0	1541.4	2.08	0.97	3.19	2.08	0.0002	0.0048
AUDd5	859.7	282.3	1437.1	2.06	0.68	3.45	2.06	0.0035	0.0259
ENTm5	1376.8	765.2	1988.4	2.06	1.14	2.97	2.06	< 0.001	0.0005
AIv6a	1281.5	386.6	2176.4	2.04	0.62	3.47	2.04	0.0050	0.0329
EPv	661.8	234.2	1089.4	2.03	0.72	3.35	2.03	0.0024	0.0203
VISp6a	689.8	274.5	1105.1	2.01	0.80	3.22	2.01	0.0011	0.0116
TEa6a	967.6	307.2	1628.1	2.00	0.63	3.36	2.00	0.0041	0.0282
SSs5	325.2	174.3	476.1	1.95	1.05	2.86	1.95	< 0.001	0.0008
SCdg	179.9	74.3	285.6	1.91	0.79	3.03	1.91	0.0008	0.0105
PERI6a	812.7	455.8	1169.6	1.89	1.06	2.73	1.89	< 0.001	0.0005
APN	176.2	62.1	290.4	1.89	0.67	3.11	1.89	0.0025	0.0203
AIp6a	1016.7	353.5	1679.8	1.85	0.64	3.06	1.85	0.0027	0.0210
SSs6a	181.6	71.2	291.9	1.83	0.72	2.95	1.83	0.0013	0.0125
AUDv5	1224.7	259.4	2190.0	1.82	0.38	3.25	1.82	0.0129	0.0647
ENTl6a	1305.7	271.4	2340.1	1.80	0.37	3.23	1.80	0.0134	0.0647
TEa6b	639.5	327.9	951.1	1.80	0.92	2.67	1.80	0.0001	0.0018
AUDpo5	974.2	409.8	1538.6	1.74	0.73	2.76	1.74	0.0007	0.0099
VISpl6b	833.1	142.0	1524.3	1.71	0.29	3.13	1.71	0.0181	0.0807
VISpl5	1794.5	315.7	3273.3	1.68	0.30	3.07	1.68	0.0174	0.0787
ACB	424.4	140.5	708.4	1.68	0.55	2.80	1.68	0.0034	0.0259
LA	394.8	179.6	609.9	1.67	0.76	2.59	1.67	0.0003	0.0059
PIL	504.0	195.9	812.2	1.67	0.65	2.69	1.67	0.0013	0.0128
BMA	733.5	197.5	1269.4	1.64	0.44	2.84	1.64	0.0073	0.0450
VISpor5	1719.9	388.1	3051.8	1.64	0.37	2.90	1.64	0.0114	0.0605
MGm	122.5	29.3	215.7	1.62	0.39	2.85	1.62	0.0100	0.0561
RSPagl6a	1031.4	117.0	1945.9	1.61	0.18	3.04	1.61	0.0270	0.1066
VISam6b	124.2	29.8	218.5	1.61	0.39	2.83	1.61	0.0099	0.0561
AUDv6a	679.2	96.8	1261.7	1.57	0.22	2.91	1.57	0.0223	0.0926
BLAp	638.0	259.8	1016.1	1.56	0.64	2.49	1.56	0.0009	0.0113
PRC	907.6	282.4	1532.8	1.55	0.48	2.62	1.55	0.0044	0.0299
VISpor6b	1289.5	182.0	2397.0	1.55	0.22	2.88	1.55	0.0225	0.0926
CEAm	122.1	6.6	237.6	1.51	0.08	2.94	1.51	0.0383	0.1374
ACAv6a	821.0	288.6	1353.3	1.51	0.53	2.49	1.51	0.0025	0.0203
LHA	360.8	128.1	593.6	1.51	0.54	2.48	1.51	0.0024	0.0203
VISpm5	1238.3	58.3	2418.2	1.50	0.07	2.93	1.50	0.0397	0.1382
DR	480.5	76.2	884.8	1.49	0.24	2.75	1.49	0.0198	0.0855
VISpor6a	719.3	179.5	1259.2	1.48	0.37	2.59	1.48	0.0090	0.0530
VISrl5	414.5	69.8	759.3	1.47	0.25	2.69	1.47	0.0184	0.0808
PMd	500.7	112.9	888.6	1.47	0.33	2.60	1.47	0.0114	0.0605
VISam5	1107.9	406.7	1809.0	1.47	0.54	2.39	1.47	0.0020	0.0180
CA1	162.2	80.0	244.3	1.46	0.72	2.19	1.46	0.0001	0.0025

Continued on next page

structure	β	β_{lower}	β_{upper}	d	d_{lower}	d_{upper}	$\lvert d \rvert$	p	q
CUN	301.8	121.5	482.0	1.44	0.58	2.30	1.44	0.0010	0.0115
TEa5	1165.0	580.2	1749.8	1.42	0.71	2.13	1.42	0.0001	0.0024
VISa5	630.4	116.3	1144.5	1.41	0.26	2.56	1.41	0.0162	0.0773
MT	158.4	16.7	300.0	1.41	0.15	2.67	1.41	0.0284	0.1105
CA3	130.9	41.7	220.1	1.40	0.45	2.36	1.40	0.0040	0.0282
PA	383.9	68.4	699.5	1.33	0.24	2.42	1.33	0.0171	0.0787
POL	126.9	10.4	243.3	1.32	0.11	2.53	1.32	0.0328	0.1223
COApm	268.9	74.6	463.1	1.29	0.36	2.22	1.29	0.0067	0.0429
LT	212.5	33.2	391.7	1.27	0.20	2.35	1.27	0.0201	0.0855
CENT3	52.7	17.3	88.2	1.26	0.41	2.11	1.26	0.0036	0.0259
MEA	242.0	29.7	454.4	1.25	0.15	2.34	1.25	0.0255	0.1035
PH	608.8	163.7	1053.8	1.24	0.33	2.15	1.24	0.0073	0.0450
CL	184.5	18.1	351.0	1.24	0.12	2.35	1.24	0.0298	0.1142
SNr	112.0	19.8	204.2	1.21	0.21	2.21	1.21	0.0173	0.0787
MMme	575.2	51.8	1098.6	1.19	0.11	2.27	1.19	0.0312	0.1181
PVH	526.6	11.3	1041.9	1.14	0.02	2.26	1.14	0.0452	0.1539
PVHd	558.3	127.1	989.5	1.10	0.25	1.95	1.10	0.0112	0.0605
RSPv6a	276.8	17.0	536.6	1.07	0.07	2.08	1.07	0.0368	0.1341
CENT2	48.0	10.0	86.0	1.04	0.22	1.87	1.04	0.0134	0.0647
VISC5	350.0	74.0	626.0	1.04	0.22	1.86	1.04	0.0129	0.0647
SSp-bfd5	147.3	36.7	258.0	1.02	0.25	1.78	1.02	0.0090	0.0530
P5	41.1	18.5	63.7	0.97	0.43	1.50	0.97	0.0004	0.0064
CA2	133.2	57.3	209.1	0.93	0.40	1.46	0.93	0.0006	0.0084
ENTl3	670.0	40.7	1299.4	0.87	0.05	1.68	0.87	0.0369	0.1341
VISli6a	285.7	3.7	567.7	0.82	0.01	1.64	0.82	0.0471	0.1565
SSp-bfd6b	52.8	11.3	94.2	0.81	0.17	1.44	0.81	0.0126	0.0647
RSPagl1	-2281.9	-4451.0	-112.8	-1.09	-2.13	-0.05	1.09	0.0392	0.1382
SSp-n1	-536.5	-1008.9	-64.1	-1.18	-2.22	-0.14	1.18	0.0260	0.1040
IPI	-292.3	-579.8	-4.9	-1.41	-2.79	-0.02	1.41	0.0462	0.1556

Table S7. Sex differences (1h, $\lvert d \rvert > 0.8$). Structures that exhibited a large interaction effect of treatment and sex at baseline (1h). Sorted by standard effect size (d). Abbreviations used can be found at atlas.brain-map.org

structure	β	β_{lower}	β_{upper}	d	d_{lower}	d_{upper}	$\lvert d \rvert$	p	q
PRC	-2366.3	-2722.6	-2009.9	-4.04	-4.65	-3.43	4.04	< 0.001	< 0.001
EPd	-739.8	-919.5	-560.0	-3.84	-4.77	-2.91	3.84	< 0.001	< 0.001
PVT	-1853.1	-2344.0	-1362.2	-3.74	-4.74	-2.75	3.74	< 0.001	< 0.001
AUDp6b	-520.7	-634.2	-407.2	-3.56	-4.34	-2.79	3.56	< 0.001	< 0.001
ProS	-557.4	-741.8	-373.1	-3.47	-4.62	-2.32	3.47	< 0.001	< 0.001
ACB	-864.2	-1051.4	-677.0	-3.41	-4.15	-2.67	3.41	< 0.001	< 0.001
BMA	-1421.0	-1785.3	-1056.8	-3.18	-4.00	-2.37	3.18	< 0.001	< 0.001
LSv	-1955.3	-2372.7	-1537.8	-3.16	-3.84	-2.49	3.16	< 0.001	< 0.001
AUDp6a	-1475.8	-1853.6	-1098.0	-3.05	-3.83	-2.27	3.05	< 0.001	< 0.001
LA	-714.7	-883.2	-546.2	-3.03	-3.75	-2.32	3.03	< 0.001	< 0.001
VISpl6a	-1146.3	-1473.2	-819.4	-3.01	-3.87	-2.15	3.01	< 0.001	< 0.001
AUDv6a	-1276.3	-1705.7	-846.8	-2.94	-3.93	-1.95	2.94	< 0.001	< 0.001
VISpl6b	-1420.1	-2168.7	-671.6	-2.91	-4.45	-1.38	2.91	0.0002	0.0014
PH	-1391.5	-1807.9	-975.2	-2.84	-3.69	-1.99	2.84	< 0.001	< 0.001
SPFm	-1977.0	-2549.2	-1404.8	-2.77	-3.57	-1.97	2.77	< 0.001	< 0.001
BST	-653.1	-815.4	-490.8	-2.77	-3.46	-2.08	2.77	< 0.001	< 0.001

Continued on next page

structure	β	β_{lower}	β_{upper}	d	d_{lower}	d_{upper}	$|d|$	p	q
TEa6a	-1339.8	-2153.0	-526.6	-2.76	-4.44	-1.09	2.76	0.0012	0.0069
MEA	-534.8	-747.5	-322.0	-2.76	-3.85	-1.66	2.76	< 0.001	< 0.001
PIL	-816.2	-1056.3	-576.2	-2.71	-3.50	-1.91	2.71	< 0.001	< 0.001
VLPO	-907.4	-1232.7	-582.0	-2.49	-3.38	-1.60	2.49	< 0.001	< 0.001
APN	-228.1	-339.9	-116.3	-2.45	-3.64	-1.25	2.45	0.0001	0.0005
VISam6b	-187.6	-229.6	-145.7	-2.43	-2.97	-1.89	2.43	< 0.001	< 0.001
CL	-360.5	-503.6	-217.3	-2.42	-3.38	-1.46	2.42	< 0.001	< 0.001
VISp6a	-828.3	-1083.3	-573.3	-2.42	-3.16	-1.67	2.42	< 0.001	< 0.001
SSs6a	-237.5	-325.9	-149.1	-2.40	-3.29	-1.50	2.40	< 0.001	< 0.001
DG-mo	-213.3	-380.9	-45.7	-2.39	-4.26	-0.51	2.39	0.0126	0.0536
CEAm	-190.5	-322.3	-58.7	-2.36	-3.99	-0.73	2.36	0.0046	0.0216
CP	-137.9	-180.3	-95.6	-2.33	-3.05	-1.62	2.33	< 0.001	< 0.001
PA	-670.7	-926.0	-415.4	-2.32	-3.21	-1.44	2.32	< 0.001	< 0.001
PVH	-1054.7	-1626.4	-483.0	-2.29	-3.53	-1.05	2.29	0.0003	0.0020
LHA	-545.7	-795.3	-296.1	-2.28	-3.32	-1.24	2.28	< 0.001	0.0002
NDB	-474.3	-735.0	-213.6	-2.25	-3.49	-1.01	2.25	0.0004	0.0023
SCdg	-210.5	-298.9	-122.1	-2.23	-3.17	-1.29	2.23	< 0.001	< 0.001
AUDpo6b	-468.7	-667.0	-270.4	-2.16	-3.07	-1.25	2.16	< 0.001	< 0.001
PMd	-728.9	-1143.9	-313.9	-2.13	-3.35	-0.92	2.13	0.0006	0.0035
BLAp	-861.0	-1320.8	-401.2	-2.11	-3.24	-0.98	2.11	0.0002	0.0017
PT	-563.0	-733.6	-392.4	-2.10	-2.74	-1.47	2.10	< 0.001	< 0.001
CLA	-1007.4	-1515.2	-499.7	-2.08	-3.13	-1.03	2.08	0.0001	0.0007
EPv	-676.2	-1009.3	-343.0	-2.08	-3.10	-1.05	2.08	0.0001	0.0005
STN	-278.0	-454.7	-101.3	-2.05	-3.35	-0.75	2.05	0.0020	0.0104
AIp6a	-1107.3	-1580.5	-634.1	-2.01	-2.87	-1.15	2.01	< 0.001	< 0.001
ACAv6b	-461.9	-741.9	-181.9	-2.01	-3.23	-0.79	2.01	0.0012	0.0069
COApm	-418.6	-720.0	-117.3	-2.01	-3.45	-0.56	2.01	0.0065	0.0298
MD	-150.3	-216.0	-84.6	-2.00	-2.87	-1.12	2.00	< 0.001	0.0001
SUM	-1236.0	-2391.0	-81.0	-1.92	-3.72	-0.13	1.92	0.0359	0.1323
PVHd	-949.9	-1419.9	-479.9	-1.88	-2.80	-0.95	1.88	0.0001	0.0006
ACAv6a	-993.9	-1564.9	-422.9	-1.83	-2.87	-0.78	1.83	0.0006	0.0038
SI	-277.9	-468.1	-87.7	-1.78	-3.00	-0.56	1.78	0.0042	0.0199
RSPagl6a	-1128.5	-2216.5	-40.4	-1.77	-3.47	-0.06	1.77	0.0421	0.1528
MEPO	-526.6	-880.4	-172.7	-1.76	-2.94	-0.58	1.76	0.0035	0.0171
CUN	-368.0	-577.6	-158.3	-1.76	-2.76	-0.76	1.76	0.0006	0.0035
MGm	-131.8	-205.6	-58.1	-1.74	-2.72	-0.77	1.74	0.0005	0.0029
ILA6a	-1263.4	-1995.6	-531.3	-1.72	-2.72	-0.72	1.72	0.0007	0.0041
ORBl6a	-342.4	-469.4	-215.4	-1.72	-2.36	-1.08	1.72	< 0.001	< 0.001
AUDpo5	-944.8	-1649.3	-240.3	-1.69	-2.95	-0.43	1.69	0.0086	0.0388
COApl	-518.8	-938.4	-99.3	-1.68	-3.04	-0.32	1.68	0.0154	0.0643
OT	-278.6	-465.2	-92.1	-1.67	-2.79	-0.55	1.67	0.0034	0.0168
VISrl6a	-239.3	-421.4	-57.2	-1.64	-2.89	-0.39	1.64	0.0100	0.0439
CA1	-181.8	-348.1	-15.5	-1.63	-3.13	-0.14	1.63	0.0322	0.1217
ORBm6a	-1058.5	-1937.0	-180.0	-1.63	-2.98	-0.28	1.63	0.0182	0.0750
ILA1	-309.4	-588.2	-30.6	-1.62	-3.08	-0.16	1.62	0.0296	0.1135
SCdw	-342.9	-601.4	-84.4	-1.57	-2.76	-0.39	1.57	0.0093	0.0415
RSPagl6b	-437.5	-615.5	-259.5	-1.57	-2.21	-0.93	1.57	< 0.001	< 0.001
VISam5	-1119.6	-1812.3	-426.8	-1.48	-2.40	-0.56	1.48	0.0015	0.0082
IMD	-156.5	-311.4	-1.5	-1.47	-2.93	-0.01	1.47	0.0478	0.1671
AUDd5	-600.8	-1185.4	-16.2	-1.44	-2.84	-0.04	1.44	0.0440	0.1557
RSPv5	-500.9	-770.0	-231.9	-1.44	-2.21	-0.67	1.44	0.0003	0.0018
PL5	-1251.3	-2015.8	-486.8	-1.43	-2.31	-0.56	1.43	0.0013	0.0072

Continued on next page

structure	β	β_{lower}	β_{upper}	d	d_{lower}	d_{upper}	$\lvert d \rvert$	p	q
PPT	-529.4	-789.6	-269.1	-1.43	-2.13	-0.73	1.43	0.0001	0.0005
PIR	-468.3	-762.3	-174.2	-1.42	-2.31	-0.53	1.42	0.0018	0.0094
MMme	-631.5	-1042.3	-220.7	-1.30	-2.15	-0.46	1.30	0.0026	0.0130
ENTm5	-870.9	-1610.8	-130.9	-1.30	-2.41	-0.20	1.30	0.0211	0.0843
VISrl5	-367.5	-695.4	-39.5	-1.30	-2.46	-0.14	1.30	0.0281	0.1093
RSPd6a	-1006.7	-1858.8	-154.6	-1.27	-2.35	-0.20	1.27	0.0206	0.0835
MPN	-608.3	-1199.1	-17.5	-1.23	-2.43	-0.04	1.23	0.0436	0.1557
AVP	-495.2	-950.9	-39.5	-1.21	-2.32	-0.10	1.21	0.0332	0.1238
SSs6b	-93.6	-167.0	-20.2	-1.17	-2.09	-0.25	1.17	0.0125	0.0536
AUDv4	1379.3	8.2	2750.4	1.06	0.01	2.11	1.06	0.0486	0.1678
IPI	308.0	38.4	577.6	1.48	0.19	2.78	1.48	0.0251	0.0991
ISN	138.4	84.2	192.6	2.39	1.45	3.32	2.39	< 0.001	< 0.001

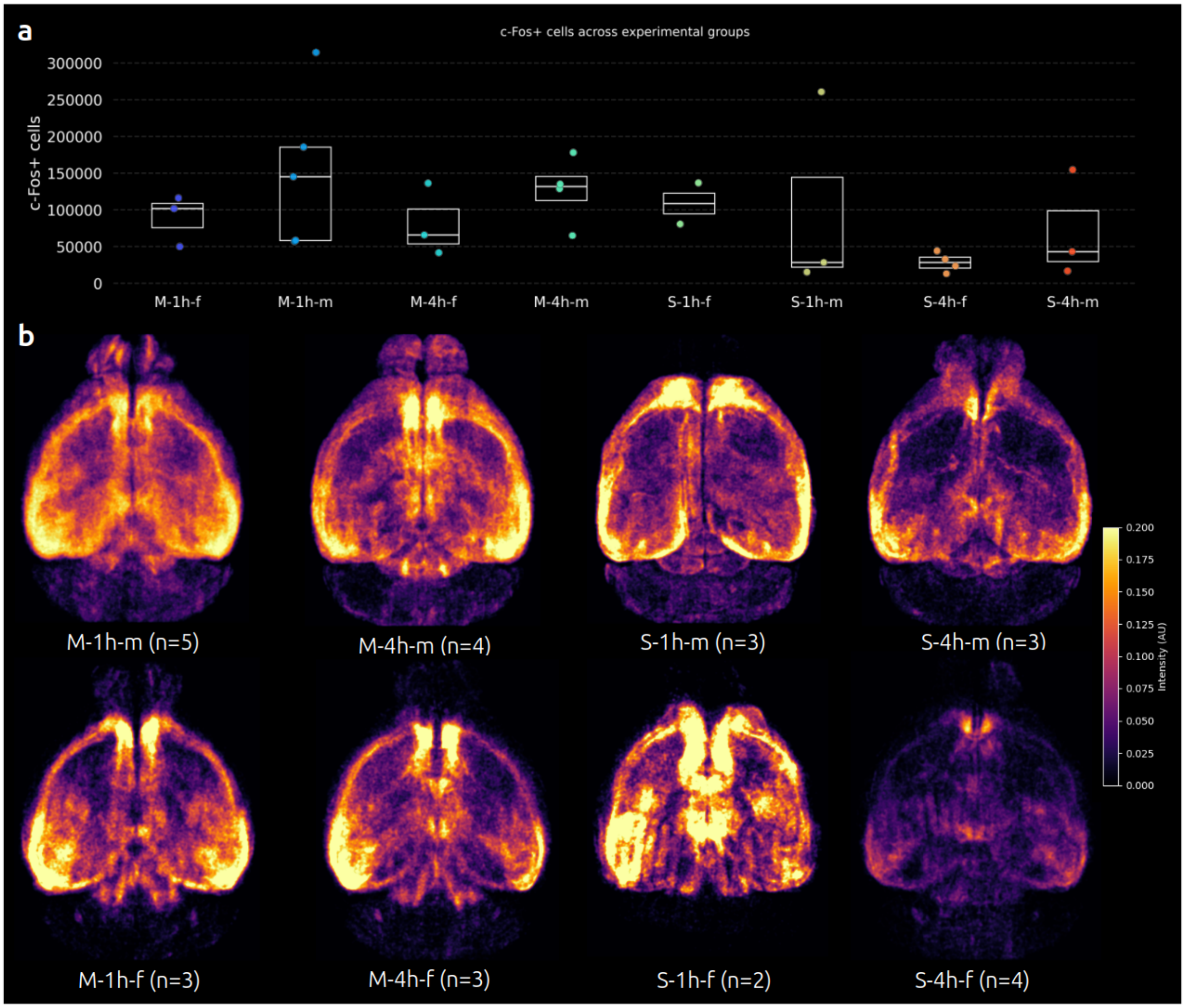

Figure S2. Temporally and sexually dimorphic activation patterns after morphine exposure. (a) Counts of c-Fos+ cells for 8 experimental groups (27 brains total): Morphine-1hour-female group (M-1h-f, average c-Fos+ density 176±40 cell/mm^3), Morphine-1hour-male group (M-1h-m, 301±94 cell/mm^3), Morphine-4hour-female group (M-4h-f, 161±56 cell/mm^3), Morphine-4hour-male group (M-4h-m, 250±46 cell/mm^3), Saline-1hour-female (S-1h-f, 215±55 cell/mm^3), Saline-1hour-male (S-1h-m, 201±158 cell/mm^3), Saline-4hour-female (S-4h-f, 56±13 cell/mm^3), Saline-4hour-male (S-4h-m, 143±83 cell/mm^3) **(b)** Maximum intensity projections of c-Fos puncta locations in the CCFv3 for morphine brains normalized (divided) by the number of brains in each group (smoothed with Gaussian filter, kernel size=4)

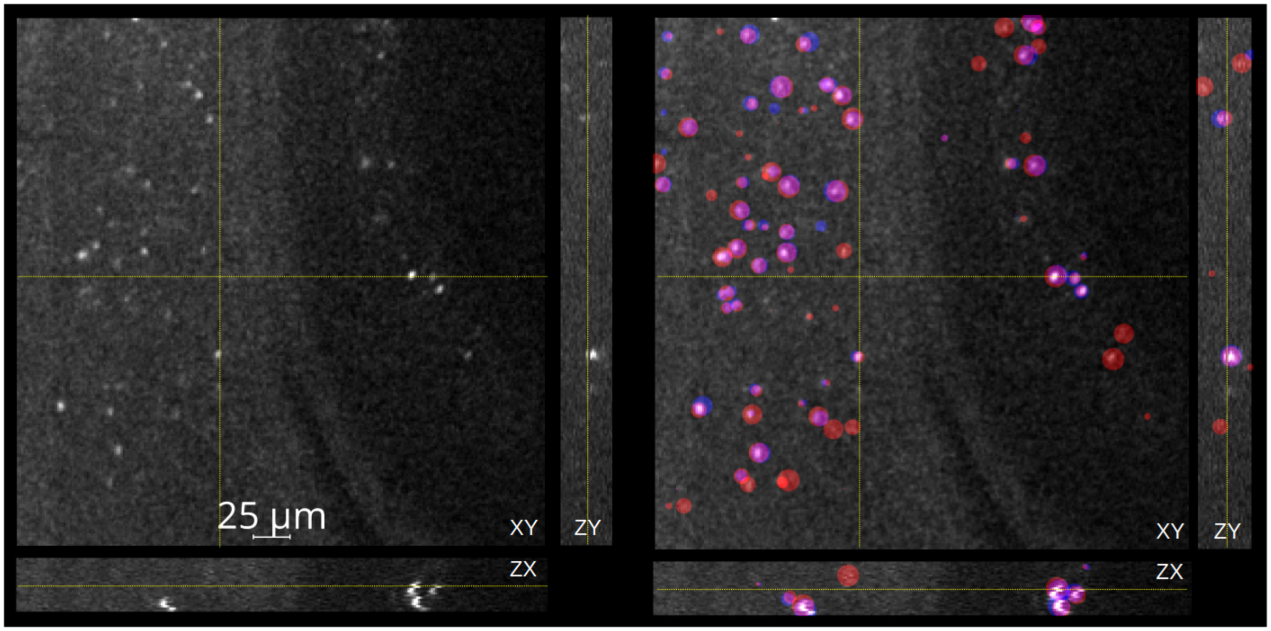

Figure S3. **c-Fos puncta detection in orthogonal planes.** Automated cell detections (red), ground truth annotations (blue), and their intersections (purple) in a 25x250x250 (z,y,x) voxel chunk from the hippocampal region. Three orthogonal planes are shown. The zx and zy planes are shown without rescaling to the isotropic resolution. The largest filled circles represent cells with centroids located within the current plane. The smaller spots also include cells where the centroid was located outside of the displayed plane, with size of the circle corresponding to the distance (in z) from the centroid to the displayed plane. High numeric aperture confocal systems like RSCM suffer from spherical aberration which causes the c-Fos puncta to be detected in several z-layers, but the algorithm prevents double-counting by using DBSCAN clustering.

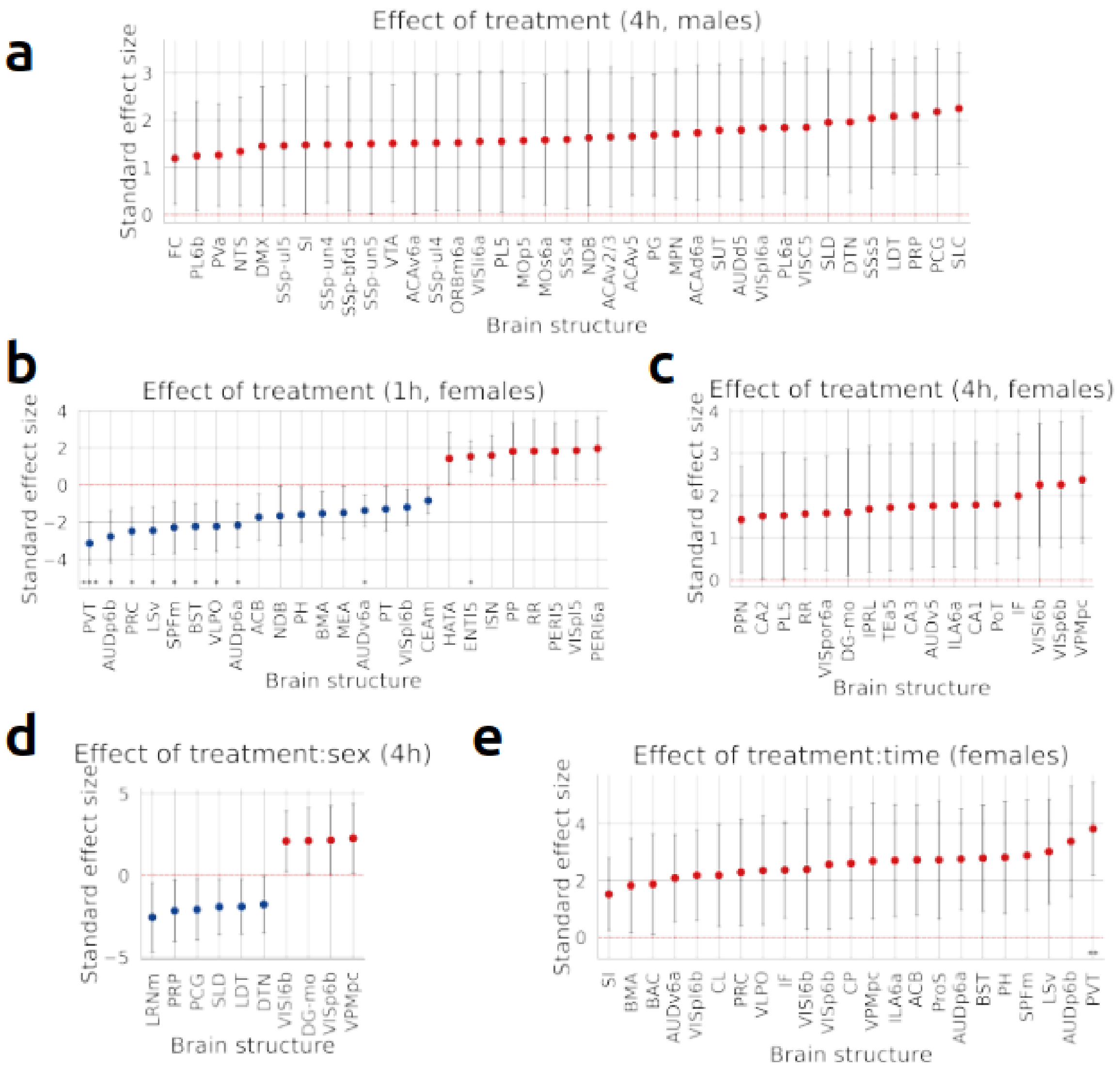

Figure S4. Non-baseline effects. (a) Structures that exhibit large effect ($|d| > 0.8$) of treatment in males at 4h. **(b)** Structures that exhibit large effect of treatment in females at 1h. **(c)** Structures that exhibit large effect of treatment in females at 4h. **(d)** Structures having sex differences at 4h. **(e)** Structures having time differences in females. Structures with $q < 0.05$ are marked with standardized star notation (* $q < 0.05$, ** $q < 0.01$, *** $q < 0.001$). Abbreviations used can be found at atlas.brain-map.org or in the list of abbreviations above

Table S2. Morphine responsive structures (4h, male, $|d| > 0.8$) Sorted by standard effect size (d). Abbreviations used can be found at atlas.brain-map.org

structure	β	β_{lower}	β_{upper}	d	d_{lower}	d_{upper}	$\|d\|$	p	q
SLC	1683.2	800.9	2565.6	2.25	1.07	3.43	2.25	0.0002	0.0510
PCG	652.9	256.0	1049.8	2.18	0.85	3.50	2.18	0.0013	0.0697
PRP	142.7	58.6	226.9	2.10	0.86	3.34	2.10	0.0009	0.0612
LDT	840.0	350.3	1329.7	2.08	0.87	3.30	2.08	0.0008	0.0612
SSs5	340.1	93.3	586.9	2.04	0.56	3.52	2.04	0.0069	0.2587
DTN	301.3	72.1	530.4	1.96	0.47	3.45	1.96	0.0100	0.2587
SLD	1440.3	616.1	2264.6	1.95	0.84	3.07	1.95	0.0006	0.0612
VISC5	622.1	119.4	1124.9	1.85	0.35	3.34	1.85	0.0153	0.2587
PL6a	1386.5	343.1	2429.9	1.84	0.46	3.23	1.84	0.0092	0.2587
VISpl6a	699.9	139.5	1260.3	1.84	0.37	3.31	1.84	0.0144	0.2587
AUDd5	747.2	124.1	1370.2	1.79	0.30	3.29	1.79	0.0187	0.2587
SUT	337.1	73.4	600.7	1.79	0.39	3.19	1.79	0.0122	0.2587
ACAd6a	1273.4	220.5	2326.2	1.73	0.30	3.17	1.73	0.0178	0.2587
MPN	845.0	164.7	1525.2	1.71	0.33	3.09	1.71	0.0149	0.2587
PG	528.6	125.1	932.2	1.68	0.40	2.97	1.68	0.0102	0.2587
ACAv5	821.2	200.8	1441.6	1.65	0.40	2.90	1.65	0.0095	0.2587
ACAv2/3	727.7	73.4	1382.0	1.65	0.17	3.13	1.65	0.0293	0.2992
NDB	343.5	40.3	646.7	1.63	0.19	3.07	1.63	0.0264	0.2802
SSs4	443.7	37.0	850.3	1.59	0.13	3.05	1.59	0.0325	0.3203
MOs6a	483.9	64.5	903.2	1.58	0.21	2.95	1.58	0.0237	0.2777
MOp5	327.5	75.9	579.2	1.57	0.36	2.78	1.57	0.0107	0.2587
PL5	1354.5	47.9	2661.1	1.55	0.05	3.05	1.55	0.0422	0.3423
VISli6a	537.7	24.1	1051.4	1.55	0.07	3.03	1.55	0.0402	0.3359
ORBm6a	990.8	50.9	1930.7	1.52	0.08	2.97	1.52	0.0388	0.3359
SSp-ul4	175.7	10.1	341.3	1.52	0.09	2.95	1.52	0.0376	0.3359
ACAv6a	824.4	12.8	1636.0	1.51	0.02	3.00	1.51	0.0465	0.3614
VTA	363.0	64.9	661.1	1.51	0.27	2.75	1.51	0.0170	0.2587
SSp-un5	265.5	3.3	527.8	1.50	0.02	2.99	1.50	0.0472	0.3614
SSp-bfd5	215.4	10.3	420.4	1.49	0.07	2.90	1.49	0.0395	0.3359
SSp-un4	234.1	39.2	429.0	1.49	0.25	2.72	1.49	0.0185	0.2587
SI	229.9	1.6	458.2	1.48	0.01	2.94	1.48	0.0484	0.3614
SSp-ul5	478.7	59.7	897.7	1.46	0.18	2.74	1.46	0.0251	0.2777
DMX	829.5	105.3	1553.8	1.45	0.18	2.72	1.45	0.0248	0.2777
NTS	189.1	25.6	352.6	1.34	0.18	2.49	1.34	0.0234	0.2777
PVa	1476.9	208.2	2745.6	1.26	0.18	2.35	1.26	0.0225	0.2777
PL6b	924.3	71.2	1777.4	1.25	0.10	2.40	1.25	0.0337	0.3209
FC	256.7	46.7	466.7	1.19	0.22	2.16	1.19	0.0166	0.2587

Table S3. Morphine responsive structures (1h, female, |d| > 0.8). Sorted by standard effect size (d). Abbreviations used can be found at atlas.brain-map.org

structure	β	β_{lower}	β_{upper}	d	d_{lower}	d_{upper}	|d|	p	q
PVT	-1556.9	-2120.3	-993.4	-3.15	-4.28	-2.01	3.15	< 0.001	< 0.001
AUDp6b	-407.3	-610.7	-203.9	-2.79	-4.18	-1.40	2.79	0.0001	0.0117
PRC	-1458.6	-2204.7	-712.6	-2.49	-3.77	-1.22	2.49	0.0001	0.0117
LSv	-1520.3	-2319.4	-721.2	-2.46	-3.75	-1.17	2.46	0.0002	0.0133
SPFm	-1631.6	-2641.4	-621.8	-2.29	-3.70	-0.87	2.29	0.0015	0.0434
BST	-527.6	-822.4	-232.7	-2.24	-3.49	-0.99	2.24	0.0005	0.0179
VLPO	-812.6	-1310.7	-314.6	-2.23	-3.59	-0.86	2.23	0.0014	0.0434
AUDp6a	-1051.3	-1623.5	-479.0	-2.17	-3.36	-0.99	2.17	0.0003	0.0175
ACB	-439.8	-756.8	-122.8	-1.74	-2.99	-0.48	1.74	0.0066	0.1507
NDB	-352.3	-688.3	-16.3	-1.67	-3.26	-0.08	1.67	0.0399	0.4588
PH	-782.8	-1501.6	-63.9	-1.60	-3.07	-0.13	1.60	0.0328	0.4314
BMA	-687.6	-1205.6	-169.6	-1.54	-2.70	-0.38	1.54	0.0093	0.1970
MEA	-292.7	-568.2	-17.3	-1.51	-2.93	-0.09	1.51	0.0373	0.4470
AUDv6a	-597.1	-967.2	-226.9	-1.38	-2.23	-0.52	1.38	0.0016	0.0434
PT	-347.9	-667.1	-28.7	-1.30	-2.49	-0.11	1.30	0.0327	0.4314
VISpl6b	-587.0	-1056.6	-117.3	-1.20	-2.17	-0.24	1.20	0.0143	0.2819
CEAm	-68.4	-125.9	-10.9	-0.85	-1.56	-0.14	0.85	0.0197	0.3392
HATA	710.8	21.8	1399.8	1.42	0.04	2.80	1.42	0.0432	0.4766
ENTl5	728.9	326.6	1131.3	1.54	0.69	2.39	1.54	0.0004	0.0177
ISN	92.3	29.2	155.4	1.59	0.50	2.68	1.59	0.0042	0.1041
PP	343.5	51.8	635.1	1.81	0.27	3.34	1.81	0.0210	0.3407
RR	303.6	20.7	586.5	1.82	0.12	3.52	1.82	0.0354	0.4443
PERI5	1266.0	223.9	2308.2	1.82	0.32	3.32	1.82	0.0173	0.3176
VISpl5	1975.5	264.7	3686.3	1.85	0.25	3.45	1.85	0.0236	0.3431
PERI6a	841.2	119.9	1562.4	1.96	0.28	3.64	1.96	0.0223	0.3414

Table S4. Morphine responsive structures (4h, female, |d| > 0.8). Sorted by standard effect size (d). Abbreviations used can be found at atlas.brain-map.org

structure	β	β_{lower}	β_{upper}	d	d_{lower}	d_{upper}	|d|	p	q
PPN	180.7	21.2	340.1	1.43	0.17	2.69	1.43	0.0264	0.5281
CA2	217.3	3.4	431.2	1.51	0.02	3.00	1.51	0.0465	0.7546
PL5	1333.4	30.5	2636.3	1.53	0.03	3.02	1.53	0.0449	0.7546
RR	261.3	42.0	480.6	1.57	0.25	2.88	1.57	0.0195	0.5281
VISpor6a	769.3	114.7	1423.8	1.58	0.24	2.93	1.58	0.0213	0.5281
DG-mo	142.6	9.0	276.2	1.60	0.10	3.09	1.60	0.0365	0.6709
IPRL	1170.9	134.6	2207.1	1.68	0.19	3.17	1.68	0.0268	0.5281
TEa5	1409.0	178.4	2639.7	1.71	0.22	3.21	1.71	0.0248	0.5281
CA3	162.6	23.2	302.0	1.74	0.25	3.24	1.74	0.0222	0.5281
AUDv5	1183.3	200.4	2166.2	1.75	0.30	3.21	1.75	0.0183	0.5281
ILA6a	1302.4	219.4	2385.3	1.77	0.30	3.25	1.77	0.0184	0.5281
CA1	198.1	31.4	364.8	1.78	0.28	3.28	1.78	0.0198	0.5281
PoT	134.4	28.1	240.7	1.79	0.38	3.21	1.79	0.0132	0.5281
IF	794.0	204.9	1383.1	1.99	0.51	3.47	1.99	0.0082	0.5281
VISl6b	760.5	270.7	1250.4	2.25	0.80	3.71	2.25	0.0023	0.2791
VISp6b	469.7	159.1	780.2	2.26	0.76	3.75	2.26	0.0030	0.2791
VPMpc	219.5	81.3	357.7	2.38	0.88	3.87	2.38	0.0019	0.2791

Table S5. Time differences (males, |d| > 0.8). Structures that exhibited a large interaction effect of treatment and time at baseline (in male mice). Sorted by standard effect size (d). Abbreviations used can be found at atlas.brain-map.org

structure	β	β_{lower}	β_{upper}	d	d_{lower}	d_{upper}	$\lvert d \rvert$	p	q
PRP	178.3	95.6	260.9	2.62	1.41	3.84	2.62	< 0.001	0.0033
PCG	627.8	7.7	1247.9	2.09	0.03	4.16	2.09	0.0472	0.5392
SLC	1391.5	932.4	1850.5	1.86	1.25	2.47	1.86	< 0.001	< 0.001
SSp-bfd4	368.9	45.6	692.3	1.83	0.23	3.43	1.83	0.0253	0.3885
SSs4	468.7	67.0	870.5	1.68	0.24	3.13	1.68	0.0222	0.3831
LDT	650.9	139.0	1162.8	1.61	0.34	2.88	1.61	0.0127	0.3489
PG	488.6	107.9	869.3	1.56	0.34	2.77	1.56	0.0119	0.3489
NTS	199.9	20.9	379.0	1.41	0.15	2.68	1.41	0.0286	0.4161
PVa	1437.5	295.1	2579.9	1.23	0.25	2.20	1.23	0.0137	0.3489
FC	258.9	87.2	430.5	1.20	0.40	2.00	1.20	0.0031	0.1722
MOp5	238.4	85.0	391.8	1.14	0.41	1.88	1.14	0.0023	0.1722
PL6b	749.6	93.9	1405.2	1.01	0.13	1.90	1.01	0.0250	0.3885
LAV	-145.0	-265.2	-24.7	-0.98	-1.80	-0.17	0.98	0.0182	0.3831
VISpm2/3	-1973.7	-3895.8	-51.6	-1.17	-2.30	-0.03	1.17	0.0442	0.5392
POL	-146.7	-287.6	-5.9	-1.53	-2.99	-0.06	1.53	0.0412	0.5392
LP	-163.5	-319.4	-7.7	-1.93	-3.77	-0.09	1.93	0.0397	0.5392
TEa6b	-698.9	-1262.2	-135.6	-1.96	-3.55	-0.38	1.96	0.0150	0.3489
APN	-194.3	-385.0	-3.6	-2.08	-4.13	-0.04	2.08	0.0458	0.5392
VISp5	-997.0	-1801.8	-192.3	-2.11	-3.81	-0.41	2.11	0.0152	0.3489
VISam6b	-170.0	-315.4	-24.6	-2.20	-4.09	-0.32	2.20	0.0219	0.3831
PP	-440.8	-811.2	-70.3	-2.32	-4.27	-0.37	2.32	0.0197	0.3831
PRC	-1499.8	-2594.8	-404.7	-2.56	-4.43	-0.69	2.56	0.0073	0.3343
DG-mo	-233.1	-416.8	-49.3	-2.61	-4.67	-0.55	2.61	0.0129	0.3489
EPd	-581.3	-962.0	-200.6	-3.02	-4.99	-1.04	3.02	0.0028	0.1722

Table S6. Time differences (females, |*d*| > 0.8). Structures that exhibited a large interaction effect of treatment and time in female mice. Sorted by standard effect size (*d*). Abbreviations used can be found at atlas.brain-map.org

structure	β	β_{lower}	β_{upper}	d	d_{lower}	d_{upper}	$\lvert d \rvert$	p	q
PVT	1886.3	1081.0	2691.6	3.81	2.18	5.44	3.81	< 0.001	0.0012
AUDp6b	491.7	207.6	775.9	3.37	1.42	5.31	3.37	0.0007	0.0959
LSv	1859.8	723.0	2996.6	3.01	1.17	4.85	3.01	0.0013	0.1236
SPFm	2050.2	667.0	3433.4	2.87	0.94	4.81	2.87	0.0037	0.1688
PH	1372.4	409.3	2335.4	2.80	0.84	4.77	2.80	0.0052	0.1836
BST	655.4	214.7	1096.2	2.78	0.91	4.65	2.78	0.0036	0.1688
AUDp6a	1328.9	469.9	2187.9	2.75	0.97	4.52	2.75	0.0024	0.1675
ProS	436.2	101.7	770.7	2.72	0.63	4.80	2.72	0.0106	0.1949
ACB	687.7	194.4	1181.1	2.71	0.77	4.66	2.71	0.0063	0.1836
ILA6a	1979.3	533.1	3425.6	2.70	0.73	4.66	2.70	0.0073	0.1836
VPMpc	247.4	58.9	435.9	2.68	0.64	4.72	2.68	0.0101	0.1949
CP	153.7	38.0	269.3	2.60	0.64	4.55	2.60	0.0092	0.1949
VISp6b	532.5	55.5	1009.6	2.56	0.27	4.85	2.56	0.0287	0.3770
VISl6b	804.1	89.9	1518.4	2.38	0.27	4.50	2.38	0.0273	0.3770
IF	941.9	269.3	1614.4	2.36	0.68	4.05	2.36	0.0061	0.1836
VLPO	855.9	158.4	1553.4	2.35	0.43	4.26	2.35	0.0162	0.2757
PRC	1337.1	239.2	2435.0	2.28	0.41	4.16	2.28	0.0170	0.2757
CL	324.8	55.7	594.0	2.18	0.37	3.98	2.18	0.0180	0.2758
VISpl6b	1060.4	285.4	1835.3	2.18	0.59	3.77	2.18	0.0073	0.1836
AUDv6a	900.3	234.2	1566.4	2.08	0.54	3.61	2.08	0.0081	0.1855
BAC	859.5	43.8	1675.3	1.86	0.09	3.63	1.86	0.0389	0.4669
BMA	810.1	65.2	1555.0	1.81	0.15	3.48	1.81	0.0331	0.4147
SI	235.3	37.6	433.0	1.51	0.24	2.78	1.51	0.0197	0.2858

Table S8. Sex differences (4h, |*d*| > 0.8). Structures that exhibited a large interaction effect of treatment and sex at 4h time point. Sorted by standard effect size (*d*). Abbreviations used can be found at atlas.brain-map.org

structure	β	β_{lower}	β_{upper}	d	d_{lower}	d_{upper}	$\lvert d \rvert$	p	q
LRNm	-116.8	-212.1	-21.5	-2.58	-4.69	-0.48	2.58	0.0163	0.9985
PRP	-147.9	-276.9	-18.9	-2.18	-4.08	-0.28	2.18	0.0247	0.9985
PCG	-632.0	-1196.4	-67.6	-2.11	-3.99	-0.23	2.11	0.0282	0.9985
SLD	-1435.0	-2692.7	-177.3	-1.95	-3.65	-0.24	1.95	0.0253	0.9985
LDT	-779.0	-1458.7	-99.2	-1.93	-3.62	-0.25	1.93	0.0247	0.9985
DTN	-276.3	-546.8	-5.9	-1.80	-3.56	-0.04	1.80	0.0452	0.9985
VISl6b	699.5	74.1	1325.0	2.07	0.22	3.93	2.07	0.0284	0.9985
DG-mo	187.3	7.6	367.0	2.10	0.08	4.11	2.10	0.0411	0.9985
VISp6b	443.3	2.8	883.9	2.13	0.01	4.25	2.13	0.0486	0.9985
VPMpc	207.9	12.6	403.2	2.25	0.14	4.36	2.25	0.0369	0.9985

Table S9. Alignment quality metrics. The following 4 metrics were calculated between downsampled raw data transformed to the CCF space and the atlas reference image: normalized cross-correlation (NCC) ranging from -1 to 1, with values > 0.6 considered "good", normalized mutual information (NMI) ranging from 0 to 2, with good values > 1.15 and gradient correlation (GC) ranging from 0 to 1, with good values > 0.1

brain id	NCC	NMI	GC
0833	0.68	1.10	0.12
0834	0.79	1.16	0.35
0835	0.69	1.09	0.22
0836	0.82	1.16	0.30
1093	0.80	1.14	0.18
1103	0.81	1.15	0.24
1106	0.38	1.05	0.06
1109	0.81	1.14	0.18
1111	0.85	1.17	0.29
1115	0.85	1.16	0.22
1116	0.86	1.17	0.34
1117	0.86	1.17	0.30
1151	0.81	1.15	0.30
1159	0.32	1.03	0.07
1161	0.77	1.15	0.28
1174	0.80	1.15	0.21
1175	0.71	1.10	0.10
1178	0.63	1.09	0.07
1179	0.55	1.06	0.09
1181	0.78	1.15	0.15
1182	0.67	1.09	0.10
1202	0.79	1.16	0.29
1249	0.53	1.07	0.07
1250	0.76	1.12	0.11
1251	0.72	1.16	0.30
1253	0.71	1.10	0.11
1255	0.74	1.11	0.19
